# Synthesis and Optimization of Ethylenediamine-Based Zwitterion on Polymer Side Chain for Recognizing Narrow Tumorous pH Windows

**DOI:** 10.1021/acs.biomac.4c01086

**Published:** 2024-10-31

**Authors:** Masahiro Toyoda, Yutaka Miura, Motoaki Kobayashi, Masato Tsuda, Takahiro Nomoto, Yuto Honda, Hiroyuki Nakamura, Hiroyasu Takemoto, Nobuhiro Nishiyama

**Affiliations:** † Laboratory for Chemistry and Life Science, Institute of Innovative Research, Tokyo Institute of Technology, 4259 Nagatsutacho, Midori-ku, Yokohama, Kanagawa 226-8501, Japan; ‡ Department of Life Science and Technology, School of Life Science and Technology, Tokyo Institute of Technology, 4259 Nagatsutacho, Midori-ku, Yokohama, Kanagawa 226-8501, Japan; § Innovation Center of Nanomedicine (iCONM), Kawasaki Institute of Industrial Promotion, 3-25-14 Tonomachi, Kawasaki-ku, Kawasaki, Kanagawa 210-0821, Japan; ∥ Department of Life Sciences, Graduate School of Arts and Sciences, The University of Tokyo, Tokyo, 3-8-1 Komaba, Meguro-ku, Tokyo 153-8902, Japan; ⊥ Medical Chemistry, Graduate School of Medical Science, Kyoto Prefectural University of Medicine, 1-5 Shimogamohangi-cho, Sakyo-ku, Kyoto 606-0823, Japan

## Abstract

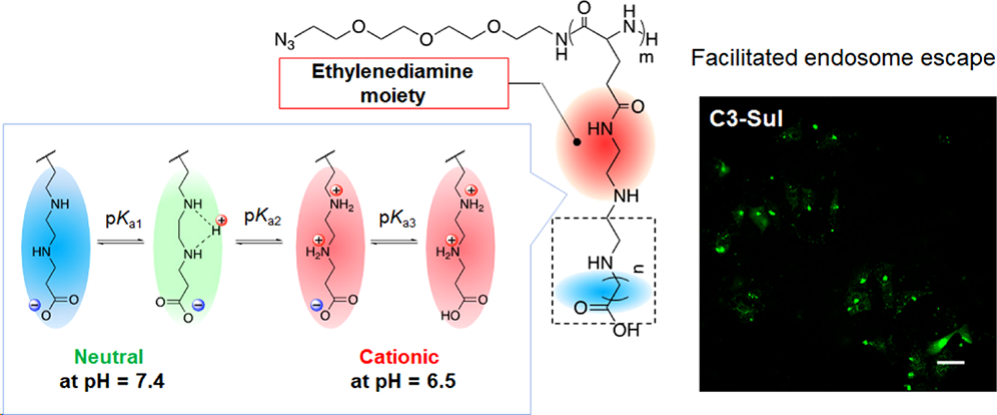

Polyzwitterions that show the alternation of net charge in response to external stimuli have attracted great attention as a new class of surface-polymers on nanomedicines. However, the correlation between their detailed molecular structures and expression of antifouling properties under physiological condition remain controversial. Herein, we synthesized a series of ethylenediamine-based polyzwitterions with carboxy groups/sulfonic groups and ethylene, propylene, and butylene spacers as potential surface-polymers for nanomedicines, allowing sensitive recognition of tumor acidic environments (pH = 6.5–5.5). Then, we evaluated their structure-based characteristics, including pH-dependent cellular uptakes and intracellular distributions. Additionally, the role of conformation stability, *i.e.*, Gibbs free energy changes, was to induce an intramolecular electrostatic interaction in the zwitterionic moieties. These results highlight the practicality of fine-tuning the design of zwitterionic moieties on polymers for the future development of nanomedicines that can recognize the narrow pH window in tumor acidic environments.

## 1 Introduction

Over the past few decades, many studies have been conducted on the development of functional polymers for drug discovery and formulation.^1−3^ Especially, polymeric surfaces on nanomedicine play a crucial role for exhibiting both antifouling properties and therapeutic effects.^1,3−5^ Among such shell polymers, polyethylene glycol (PEG) has been widely used because of its high biocompatibility and safety.^6^ However, some PEG-related issues have been highlighted in recent research. Due to daily contact with PEG-modified materials for many years in the cosmetics, food, and pharmaceutical areas, the production of anti-PEG antibodies and PEG-related immune responses were confirmed when PEG-related products or PEG-modified nanomedicines were systemically administered to the body.^6,7^ Accelerated blood clearance (ABC) phenomenon is also important, and thus the guideline and reflection papers on the use of PEGylated drugs were released by the U.S. Department of Health and Human Services Food and Drug Administration (FDA) and European Medicines Agency (EMA).^8−10^ Moreover, PEG modification of nanomedicines often inhibits cellular uptake and endosomal escape (known as the “PEG dilemma”) due to the hindrance of the interaction between the cell membrane and PEG-modified surface, resulting in the reduction of therapeutic efficacy.^11,12^ Nucleic acid therapeutics such as small interfering RNA and mRNA are becoming the mainstream of drug discovery, and they need to be delivered into the cytoplasm of targeted cell to exhibit their therapeutic effects. Therefore, there has been a renewal of interest in surface-polymers, especially among the development of systemically injectable nanomedicines.

To overcome such disadvantages of PEG, we have previously developed the novel polyzwitterions “ethylenediamine (EDA)-based poly(carboxybetaine) poly(*N*-{*N*′-[*N*″-(2-carboxyethyl)-2-aminoethyl]-2-aminoethyl}glutamide) (PGlu(DET-Car))”.^13^ The EDA moiety in PGlu(DET-Car) is monoprotonated under physiological conditions (pH = 7.4), whereas it is diprotonated under acidic conditions.^14,15^ This pH responsiveness is attractive for tumor-targeting applications because pH of the bloodstream is 7.4, whereas the peripheries of tumor tissues are slightly acidic (pH = ∼6.5) due to the Warburg effect.^16−19^ Although the differences between the pH values of the bloodstream and the tumor microenvironment are quite narrow, these pH differences are considered to be hallmarks of solid tumors. Our PGlu(DET-Car)-functionalized nanomedicines recognized this narrow pH window: they showed antifouling properties with a neutral net charge at pH = 7.4, whereas they interacted with tissue and cellular components such as endosome with a cationic net charge at pH = 6.5, resulting in higher tumor accumulation and efficient endosome escape compared to PEG-coated systems.^13,20−22^ However, this pH responsiveness in the previous studies was demonstrated with limited chemical design, *i.e.*, the carboxy group with an ethylene carbon spacer. Since the physical properties of polyzwitterions are highly dependent on the combination of the ionic groups (e.g., carboxylic and sulfonic acid) and the lengths of carbon (C) spacer,^23−25^ the structure-based fine-tunings in polyzwitterion remain to be optimized toward the development of nanomedicines. To further develop the pH-responsive polymers as functional materials, it is important to understand the detailed influences of such structural factors on the properties of polyzwitterions and optimize the zwitterionic moiety for effective switching between the antifouling property and tumor targetability in response to the narrow pH window.

**Figure 1:**
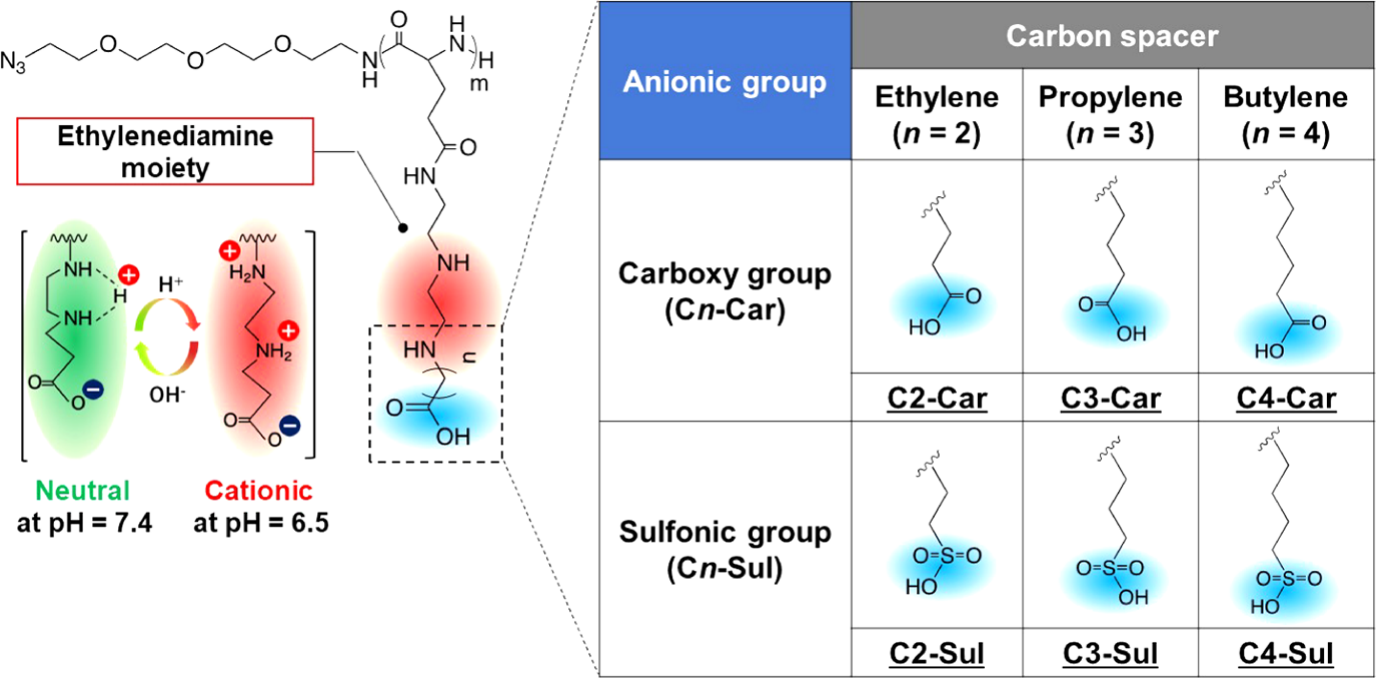
Chemical structures and abbreviations of the ethylenediamine-based polyzwitterions synthesized in this study.

In this study, we focused on the synthesis of a series of new polyzwitterions, *i.e.*, EDA-based polyzwitterions with carboxy groups (C*n*-Car) or sulfonic groups (C*n*-Sul) as negatively charged moieties and ethylene (*n* = 2), propylene (*n* = 3), and butylene (*n* = 4) spacers (Figure 1). We investigated the protonation behaviors and abilities of these polyzwitterions to interact with tissue components and cells in response to the surrounding pH. Finally, we evaluated the conformational stabilities of these polyzwitterions by using density functional theory (DFT) to explain the unique properties of these polymers. These studies highlight the utility of side-chain design for amplifying the potentials of polyzwitterions as functional materials for drug delivery application.

## 2 Materials and Methods

### 2.1 Materials

γ-Benzyl-l-glutamate *N*-carboxy anhydride (BLG-NCA) was purchased from Chuo Kaseihin Co., Ltd. (Tokyo, Japan). *N*,*N*-dimethylformamide (DMF), *N*-methyl-2-pyrrolidone (NMP), dichloromethane (DCM), methanol, ethanol, *tert*-butanol (*tert*-BuOH), triethylamine (TEA), *p*-tolualdehyde, trimethyl(trifluoromethyl)silane, fluorescein-4-isothiocyanate (FITC), sodium chloride (NaCl), sodium iodide (NaI), 4-(2-hydroxyethyl)-1-piperazineethanesulfonic acid (HEPES), 2-(*N*-morpholino)ethanesulfonic acid (MES), sodium dihydrogen phosphate, sodium bicarbonate, disodium carbonate, heparin, and tetrahydrofuran (THF) were procured from FUJIFILM Wako Pure Chemical Corporation (Osaka, Japan). DMF and DCM were distilled *in vacuo* before use. Hydrochloric acid solution (5 M), sodium hydroxide solution (5 M), Dulbecco’s phosphate buffered saline (PBS), and chloroquine diphosphate were purchased from Nacalai Tesque, Inc. (Kyoto, Japan). Deuterated chloroform (CDCl_3_) and deuterated dimethyl sulfoxide (DMSO) (DMSO-*d*_6_) were obtained from Cambridge Isotope Laboratories, Inc. (Tewksbury, MA). 3-Chloropropane sulfonyl chloride, 1,4-butane sultone, deuterium oxide (D_2_O), 11-azido-3,6,9-trioxaundecan-1-amine, and Roswell Park Memorial Institute (RPMI) 1640 medium were purchased from Sigma-Aldrich (St. Louis, MO). α-Methoxy-ω-amino-poly(ethylene glycol) (*M*_n_ = 20,000 g/mol) (MeO-PEG_20k_-NH_2_) was procured from NOF Co., Ltd. (Tokyo, Japan). *tert*-Butyl acrylate, 4-bromobutyric acid, 5-bromopentanoic acid, tetrabutylammonium fluoride (ca. 1 in THF), 2-chloroethane sulfonyl chloride, diethylenetriamine (DET), 2-hydroxypyridine (2-HP), and thionyl chloride (SOCl_2_) were purchased from Tokyo Chemical Industry Co., Ltd. (Tokyo, Japan). Cy3-dibenzocyclooctyne (DBCO) (Cy3-DBCO) was obtained from Click Chemistry Tools (Scottsdale, AZ).

### 2.2 NMR Characterization

Proton nuclear magnetic resonance (^1^H NMR) spectra were recorded using a Bruker biospin AVANCE III 400A (400 MHz) (Bruker Corporation, Billerica, MA) instrument with CDCl_3_ and CD_3_OD containing tetramethylsilane as the internal standard. All chemical shifts are given in parts per million (δ) units. The analysis followed first order, and the following abbreviations were used throughout the text: *s* = singlet, *d* = doublet, *t* = triplet, *q* = quartet, *dd* = doublet of doublets, and *m* = multiplet.

### 2.3 Electrospray Ionization Mass Spectrometry (ESI-MS) Characterization

A Bruker Daltonics micrOTOF II (Billerica, MA) instrument was used to measure electrospray ionization-time-of-flight mass (ESI-MS). The samples were dissolved in CH_3_OH and then injected into the column at room temperature (r.t.).

### 2.4 Cells

Human liver carcinoma (Huh-7) cell line was purchased from ATCC (Manassas, VA). The cells were cultured under a humidified atmosphere containing 5% CO_2_ at 37 °C in Dulbecco’s modified eagle medium (DMEM) comprising 10% fetal bovine serum (FBS) and 1% penicillin/streptomycin.

### 2.5 Synthesis of *N*-{2[(2-Aminoethyl)amino]ethyl}-3-aminopropanoic Acid *tert*-Butyl Ester (DET-C2-Car *t*Bu) (**1**)

*tert*-Butyl acrylate (10.1 g, 78.8 mmol) was added dropwise to DET (65.2 g, 0.632 mol) followed by stirring for 2 h at r.t. Then, the resulting reaction solution was mixed with DCM (300 mL) followed by washing with 150 mM NaCl aq. and drying over Na_2_SO_4_. After filtering and concentrating the resulting mixture *in vacuo*, the crude product was acquired and purified by flash column chromatography (CH_3_OH/TEA = 95:5) to obtain DET-C2-Car *t*Bu (Scheme S1a) as a colorless oil (5.6 g, yield: 31%). ^1^H NMR (CD_3_OD, 400 MHz): δ = 2.85–2.65 (*m*, 10H), 2.44 (*t*, 2H, *J* = 6.8 Hz), 1.45 (*s*, 9H). ESI-MS *m*/*z*: [M + H]^+^ Calculated for C_11_H_26_N_3_O_2_Na: 232.2025; [M + H]^+^ Found: 232.2327.

### 2.6 Preparation of *tert*-Butyl 4-Bromobutanoate (**2**)

MgSO_4_ (30 g) and H_2_SO_4_ (2 mL) were added to DCM (200 mL), and the resulting mixture was stirred for 10 min. Thereafter, 4-bromobutyric acid (10.0 g, 59.9 mmol) and *tert*-BuOH (22.2 g, 0.295 mol) were introduced into the above-mentioned mixture. The resulting reaction mixture was stirred for 3 days under an Ar atmosphere and then added dropwise to sat. NaHCO_3_ aq. for quenching. The organic phase was washed with sat. NaHCO_3_ aq. and brine followed by drying over Na_2_SO_4_ and *in vacuo*. The acquired crude product was purified by flash column chromatography (ethyl acetate (EOtAc)/hexane = 3:7) to obtain **2** (Scheme S1b) as a colorless oil (11.1 g, yield: 83%). ^1^H NMR (CDCl_3_, 400 MHz): δ = 3.46 (*t*, 2H, *J* = 6.5 Hz), 2.40 (*t*, 2H, *J* = 7.2 Hz), 2.13 (*q*, 2H, *J* = 6.9 Hz), 1.45 (*s*, 9H). ESI-MS *m*/*z*: [M + Na]^+^ Calculated for C_8_H_15_BrO_2_Na: 245.0153; [M + Na]^+^ Found: 245.0426.

### 2.7 Synthesis of *N*-{2-[(2-Aminoethyl)amino)ethyl]}-4-aminobutyric Acid *tert*-Butyl Ester (DET-C3-Car *t*Bu) (**3**)

**2** (10.0 g, 45.0 mmol) was added dropwise to a solution of DET (36.9 g, 0.359 mol) in 100 mL DCM. The reaction mixture was stirred overnight at r.t. followed by washing with 150 mM NaCl aq. and drying over Na_2_SO_4_. After filtering and concentrating the resulting mixture *in vacuo*, the crude product was acquired and purified by flash column chromatography (CH_3_OH/TEA = 95:5) to obtain **3** (Scheme S1b) as a colorless oil (3.9 g, yield: 35%). ^1^H NMR (CD_3_OD, 400 MHz): δ = 2.79–2.64 (*m*, 8H), 2.60 (*t*, 2H, *J* = 7.5 Hz), 2.28 (*t*, 2H, *J* = 7.4 Hz), 1.77 (*q*, 2H, *J* = 7.1 Hz), 1.44 (*s*, 9H). ESI-MS *m*/*z*: [M + H]^+^ Calculated for C_12_H_28_N_3_O_2_: 246.2181; [M + H]^+^ Found: 246.2447.

### 2.8 Preparation of *tert*-Butyl 5-Bromopentanoate (**4**)

MgSO_4_ (30 g) and H_2_SO_4_ (2 mL) were introduced into DCM (200 mL), followed by stirring for 10 min. Subsequently, 5-bromopentanoic acid (10.0 g, 55.2 mmol) and *tert*-BuOH (20.5 g, 0.276 mol) were added to the resulting mixture. The acquired reaction solution was stirred for 3 days under an Ar atmosphere and then added dropwise to sat. NaHCO_3_ aq. for quenching. The organic phase was washed with sat. NaHCO_3_ aq. and brine followed by drying over Na_2_SO_4_ and evaporation. The acquired crude product was purified by flash column chromatography (EtOAc/hexane = 3:7) to obtain **4** (Scheme S1c) as a colorless oil (11.8 g, yield: 90%). ^1^H NMR (CDCl_3_, 400 MHz): δ = 3.41 (*t*, 2H, *J* = 6.6 Hz), 2.25 (*t*, 2H, *J* = 7.2 Hz), 1.89 (*m*, 2H, *J* = 7.4 Hz), 1.74 (*q*, 2H, *J* = 7.4 Hz), 1.45 (*s*, 9H). ESI-MS *m*/*z*: [M + Na]^+^ Calculated for C_9_H_17_BrO_2_Na: 259.0309; [M + Na]^+^ Found: 259.0642.

### 2.9 Synthesis of *N*-{2-[(2-Aminoethyl)amino]ethyl}-5-aminopentanoic Acid *tert*-Butyl Ester (DET-C4-Car *t*Bu) (**5**)

**4** (10.0 g, 42.2 mmol) was added dropwise to a solution of DET (36.9 g, 0.359 mol) in 100 mL DCM. The resulting reaction solution was stirred overnight at r.t. followed by washing with 150 mM NaCl aq. and drying over Na_2_SO_4_. After filtering and concentrating the resulting mixture *in vacuo*, the acquired crude product was purified by flash column chromatography (CH_3_OH/TEA = 95:5) to obtain **5** (Scheme S1c) as a colorless oil (3.3 g, yield: 30%). ^1^H NMR (CD_3_OD, 400 MHz): δ = 2.78–2.63 (*m*, 8H), 2.59 (*t*, 2H, *J* = 7.2 Hz), 2.24 (*t*, 2H, *J* = 7.1 Hz), 1.67–1.48 (*m*, 4H), 1.44 (*s*, 9H). ESI-MS *m*/*z*: [M + H]^+^ Calculated for C_13_H_30_N_3_O_2_: 260.2338; [M + H]^+^ Found: 260.2657.

### 2.10 Preparation of 2,2,2-Trifluoro-1-*p*-tolyl-ethanol (**6**)

**6** was synthesized according to a previously reported method.^26^ Briefly, *p*-tolualdehyde (5.0 g, 42 mmol) and trimethyl(trifluoromethyl)silane (8.3 g, 58 mmol) were dissolved in THF (83 mL) at 0 °C; then, a catalytic amount (40 drops) of tetrabutylammonium fluoride (*ca*. 1 mol/L in THF) was added dropwise to the above-mentioned solution followed by stirring for 1 h. The resulting reaction solution was concentrated *in vacuo* and purified by flash column chromatography (hexane) to obtain the trimethylsilylated precursor as a colorless oil. This precursor was dissolved in 100 mL of 1:1 THF/1 M HCl (aq) followed by stirring overnight. Thereafter, the acquired solution was diluted with water and extracted with EtOAc. The organic phase was washed with 1 M HCl aq., water, and brine followed by drying over Na_2_SO_4_ and evaporation to obtain **6** (Scheme S2a) as a colorless oil (7.0 g, yield: 89%). ^1^H NMR (400 MHz, CDCl_3_): δ = 7.35 (*d*, 2H, *J* = 7.9 Hz), 7.21 (*d,* 2H, *J* = 7.9 Hz), 4.95 (*m*, 1H), 2.60 (*d*, 1H), 2.36 (*s*, 3H, *J* = 4.5 Hz). ^19^F NMR (376 MHz, CDCl_3_): δ = −78.4 (*d*, *J* = 6.8 Hz). ESI-MS *m*/*z*: [M + Na]^+^ Calculated for C_9_H_9_F_3_ONa: 213.0503; [M + Na]^+^ Found: 213.0492.

### 2.11 Synthesis of Vinylsulfonic Acid 2,2,2-Trifluoro-1-*p*-tolyl-ethyl Ester (**7**)

2-Chloroethane sulfonyl chloride (5.6 g, 34.2 mmol) was added dropwise to a mixture of alcohol (**6,** 5.0 g, 26.3 mmol) and TEA (5.3 g, 52.6 mmol) in 100 mL of DCM at 0 °C. Then, the resulting reaction solution was heated to r.t. followed by stirring overnight under an Ar atmosphere. Subsequently, the resulting solution was filtered, and the filtrate was washed with 1 M HCl (aq) and brine followed by drying over Na_2_SO_4_. After the resulting mixture was filtered and concentrated *in vacuo*, the acquired crude product was purified by flash column chromatography (hexane) to obtain **7** (Scheme S2b) as a colorless oil (6.8 g, yield: 92%). ^1^H NMR (CDCl_3_, 400 MHz): δ = 7.35 (*d*, 2H, *J* = 7.9 Hz), 7.24 (*d*, 2H, *J* = 7.9 Hz), 6.65–6.38 (*m*, 2H), 6.01 (*dd*, 1H), 5.62 (*q*, 1H, *J* = 6.3 Hz), 2.38 (*s*, 3H). ^19^F NMR (CDCl_3_, 376 MHz): δ = −76.0 (*d*, *J* = 6.1 Hz). ESI-MS *m*/*z*: [M + Na]^+^ Calculated for C_11_H_11_F_3_O_3_SNa: 303.0279; [M + Na]^+^ Found: 303.0625.

### 2.12 Preparation of *N*-{2-[(2-Aminoethyl)amino]ethyl}-2-aminoethanesulfonic Acid 2,2,2-Trifluoro-1-*p*-tolyl-ethyl Ester (DET-C2-Sul TFPT) (**8**)

Sulfonate ester (**7**, 6.0 g, 21.4 mmol) was added dropwise to a solution of DET (17.7 g, 0.171 mol) in 100 mL DCM. The resulting reaction solution was stirred overnight at r.t. followed by washing with 150 mM NaCl aq. and drying over Na_2_SO_4_. After filtering and concentrating the resulting mixture *in vacuo*, the acquired crude product was purified by flash column chromatography (CH_3_OH/TEA = 95:5) to obtain **8** (Scheme S2b) as a colorless oil (2.5 g, yield: 30%). ^1^H NMR (CD_3_OD, 400 MHz): δ = 7.45 (*d*, 2H, *J* = 7.9 Hz), 7.30 (*d*, 2H, *J* = 7.9 Hz), 6.06 (*q*, 2H, *J* = 6.5 Hz), 3.34 (*t*, 2H, *J* = 7.0 Hz), 3.01–2.53 (*m*, 10H), 2.38 (*s*, 3H). ^19^F NMR (CD_3_OD, 376 MHz): δ = −77.7 (*d*, *J* = 6.5 Hz). ESI-MS *m*/*z*: [M + H]^+^ Calculated for C_15_H_25_F_3_N_3_O_3_S: 384.4373; [M + H]^+^ Found: 384.3901.

### 2.13 Synthesis of 3-Chloropropanesulfonic Acid 2,2,2-Trifluoro-1-*p*-tolyl-ethyl Ester (**9**)

3-Chloropropane sulfonyl chloride (6.05 g, 34.2 mmol) was added dropwise to a mixture of **6** (5.0 g, 26.3 mmol) and TEA (5.3 g, 52.6 mmol) in 100 mL of DCM at 0 °C. Thereafter, the resulting reaction mixture was heated to r.t. and stirred overnight under an Ar atmosphere. Then, the obtained mixture was filtered, and the filtrate was washed with 1 M HCl (aq) and brine followed by drying over Na_2_SO_4_. After filtering and concentrating the resulting mixture *in vacuo*, the acquired crude product was purified by flash column chromatography (hexane/EtOAc = 95:5) to obtain **9** (Scheme S2c) as a colorless oil (7.6 g, yield: 87%). ^1^H NMR (CDCl_3_, 400 MHz): δ = 7.38 (*d*, 2H, *J* = 8.0 Hz), 7.26 (*d*, 2H, *J* = 8.0 Hz), 5.74 (*q*, 2H, *J* = 6.4 Hz), 3.62–3.53 (*m*, 2H), 3.27–3.14 (*m*, 2H), 2.39 (*s*, 3H), 2.30–2.18 (*m*, 2H). ^19^F NMR (CDCl_3_, 376 MHz): δ = −75.9 (*d*, *J* = 6.5 Hz). ESI-MS *m*/*z*: [M + Na]^+^ Calculated for C_12_H_14_ClF_3_O_3_SNa: 353.0202; [M + Na]^+^ Found: 353.0592.

### 2.14 Preparation of 3-Iodopropanesulfonic Acid 2,2,2-Trifluoro-1-*p*-tolyl-ethyl Ester (**10**)

**9** (7.0 g, 21.2 mmol) was added to a suspension of NaI (12.7 g, 84.6 mmol) in acetone (100 mL) and refluxed overnight. Then, the resulting heterogeneous mixture was filtered and concentrated *in vacuo*. The acquired crude product was mixed with DCM followed by washing with brine and drying over Na_2_SO_4_. After the resulting mixture was filtered and concentrated *in vacuo*, the achieved crude product was purified by flash column chromatography (hexane) to obtain **10** (Scheme S2c) as a pale yellow oil (7.25 g, 81%). ^1^H NMR (CDCl_3_, 400 MHz): δ = 7.39 (*d*, 2H, *J* = 7.8 Hz), 7.27 (*d*, 2H, *J* = 7.8 Hz), 5.73 (*q*, 2H, *J* = 6.4 Hz), 3.21–3.07 (*m*, 4H), 2.39 (*s*, 3H), 2.32–2.20 (*m*, 2H). ^19^F NMR (CDCl_3_, 376 MHz): δ = −75.9 (*d*, *J* = 6.5 Hz). ESI-MS *m*/*z*: [M + Na]^+^ Calculated for C_12_H_14_F_3_IO_3_SNa: 444.9558; [M + Na]^+^ Found: 444.9942.

### 2.15 Synthesis of *N*-{2-[(2-Aminoethyl)amino]ethyl}-3-aminopropanesulfonic Acid 2,2,2-Trifluoro-1-*p*-tolyl-ethyl Ester (DET-C3-Sul TFPT) (**11**)

**10** (7.0 g, 16.6 mmol) was added dropwise to a solution of DET (13.7 g, 0.133 mol) in 100 mL DCM. The resulting reaction solution was stirred overnight at r.t. followed by washing with 150 mM NaCl aq. and drying over Na_2_SO_4_. After the resulting mixture was filtered and concentrated *in vacuo*, the acquired crude product was purified by flash column chromatography (CH_3_OH/TEA = 95:5) to obtain **11** (Scheme S2c) as a colorless oil (3.0 g, yield: 45%). ^1^H NMR (CD_3_OD, 400 MHz): δ = 7.44 (*d*, 2H, *J* = 7.9 Hz), 7.30 (*d*, 2H, *J* = 7.9 Hz), 6.01 (*q*, 2H, *J* = 6.5 Hz), 3.29–3.15 (*m*, 2H), 3.66 (*t*, 2H), 2.77–2.57 (*m*, 10H), 2.38 (*s*, 3H), 1.96–1.84 (*m*, 2H). ^19^F NMR (CD_3_OD, 376 MHz): δ = −77.7 (*d*, *J* = 6.5 Hz). ESI-MS *m*/*z*: [M + H]^+^ Calculated for C_16_H_27_F_3_N_3_O_3_S: 398.1725; [M + H]^+^ Found: 398.2189.

### 2.16 Preparation of 4-Chlorobutanesulfonic Acid 2,2,2-Trifluoro-1-*p*-tolyl-ethyl Ester (**12**)

1,4-Butane sultone (15 g, 0.110 mol) was added to a mixture of SOCl_2_ (11.3 mL, 0.156 mol) and DMF (2 mL) followed by stirring under an Ar atmosphere at 70 °C for 3 days. SOCl_2_ (3 mL) was added again to the resulting mixture, followed by stirring at 70 °C for another 3 days. The acquired residue was mixed with toluene followed by evaporation. This step was repeated twice, and the residue was dried under vacuum to obtain the sulfonyl chloride precursor. This precursor (15.0 g, 78.5 mmol) was added dropwise to a solution of **6** (5.0 g, 26.3 mmol) and TEA (7.9 g, 78.5 mmol) in 100 mL of DCM at 0 °C. Thereafter, the resulting reaction mixture was heated to r.t. and stirred overnight under an Ar atmosphere. Then, the obtained mixture was filtered, and the filtrate was washed with 1 M HCl aq, water, and brine followed by drying over Na_2_SO_4_. After filtering and concentrating the resulting mixture *in vacuo*, the acquired crude product was purified by flash column chromatography (hexane) to obtain **12** (7.4 g, 82%, Scheme S2d). ^1^H NMR (CDCl_3_, 400 MHz): δ = 7.38 (*d*, 2H, *J* = 8.0 Hz), 7.26 (*d*, 2H, *J* = 8.0 Hz), 5.73 (*q*, 1H, *J* = 6.37 Hz), 3.48 (*t*, 2H, *J* = 6.2 Hz), 3.11–2.97 (*m*, 2H), 2.39 (*s*, 3H), 2.00–1.92 (*m*, 2H), 1.86–1.78 (*m*, 2H). ^19^F NMR (CDCl_3_, 376 MHz): δ = −75.9 (*d*, *J* = 6.4 Hz). ESI-MS *m*/*z*: [M + Na]^+^ Calculated for C_13_H_16_ClF_3_O_3_SNa: 367.0392; [M + Na]^+^ Found: 367.0745.

### 2.17 Fabrication of 4-Iodobutanesulfonic Acid 2,2,2-Trifluoro-1-*p*-tolyl-ethyl Ester (**13**)

**12** (7.0 g, 20.3 mmol) was added to a suspension of NaI (12.2 g, 81.2 mol) in acetone (100 mL) and refluxed overnight. Thereafter, the resulting heterogeneous mixture was filtered and concentrated *in vacuo*. The acquired crude product was mixed with DCM followed by washing with water and drying over Na_2_SO_4_. After filtering and concentrating the resulting mixture *in vacuo*, the crude product was purified by flash column chromatography (hexane) to obtain **13** (7.5 g, yield: 85%, Scheme S2d). ^1^H NMR (CDCl_3_, 400 MHz): δ = 7.39 (*d*, 2H, *J* = 8.1 Hz), 7.27 (*d*, 2H, *J* = 8.1 Hz), 5.73 (*q*, 1H, *J* = 6.37 Hz), 3.11–2.95 (*m*, 4H), 2.39 (*s*, 3H), 1.98–1.78 (*m*, 4H). ^19^F NMR (CDCl_3_, 376 MHz): δ = −75.9 (*d*, *J* = 6.5 Hz). ESI-MS *m*/*z*: [M + Na]^+^ Calculated for C_13_H_16_F_3_IO_3_SNa: 459.2192; [M + Na]^+^ Found: 459.2296.

### 2.18 Synthesis of *N*-[2-{(2-Aminoethyl)amino}ethyl]-4-aminobutanesulfonic Acid 2,2,2-Trifluoro-1-*p*-tolyl-ethyl Ester (DET-C4-Sul TFPT) (**14**)

The sulfonate ester **13** (7.0 g, 16.0 mmol) was added dropwise to a solution of DET (13.2 g, 0.128 mol) in 100 mL DCM. This reaction mixture was stirred overnight at r.t. followed by washing with 150 mM NaCl aq. and drying over Na_2_SO_4_. After filtering and concentrating the resulting mixture *in vacuo*, the acquired crude product was purified by flash column chromatography (CH_3_OH/TEA = 95:5) to obtain **14** (Scheme S2d) as a pale yellow oil (2.5 g, yield: 39%). ^1^H NMR (CD_3_OD, 400 MHz): δ = 7.44 (*d*, 2H, *J* = 8.0 Hz), 7.30 (*d*, 2H, *J* = 8.0 Hz), 6.00 (*q*, 2H, *J* = 6.5 Hz), 3.24–3.08 (*m*, 2H), 2.76–2.65 (*m*, 8H), 2.48 (*t*, 2H), 2.38 (*s*, 3H), 1.82–1.66 (*m*, 2H), 1.57–1.44 (*m*, 2H). ^19^F NMR (CD_3_OD, 376 MHz): δ = −77.7 (*d*, *J* = 6.3 Hz). ESI-MS *m*/*z*: [M + H]^+^ Calculated for C_17_H_29_F_3_N_3_O_3_S: 412.4872; [M + H]^+^ Found: 412.4328.

### 2.19 Preparation of Poly(γ-benzyl-l-glutamate) (PBLG)

PBLG was synthesized by the ring-opening polymerization of BLG-NCA using 11-azido-3,6,9-trioxaundecan-1-amine as an initiator (Scheme S3). After dissolving BLG-NCA (3.01 g, 11.4 mmol, 120 equiv) in a 30 mL mixture of DMF and DCM (1/4, *v*/*v*) under an Ar atmosphere, the initiator (19.1 μL, 95.2 μmol, 1 equiv) was added to the resulting mixture followed by stirring at r.t. for 2 days. The resulting reaction solution was poured into an excess amount of Et_2_O, and the precipitate was obtained by filtration followed by drying under vacuum to achieve a white powder (2.11 g, yield: 82.3%). Then, the obtained polymer was analyzed using size exclusion chromatography (SEC) equipped with a refractive index (RI) detector (RI 2075, JASCO, Tokyo, Japan) [columns: superAW3000 and superAW4000 (TOSOH, Tokyo, Japan) and eluent: NMP with 50 mM LiBr]. Molecular weight distribution (*M*_w_/*M*_n_) of the polymer was calculated to be 1.18.

### 2.20 Syntheses of Poly(*N*-{*N′*-[*N″*-(2-carboxyethyl)-2-aminoethyl]-2-aminoethyl}glutamide) (C2-Car), Poly(*N*-{*N′*-[*N″*-(3-carboxypropyl)-2-aminoethyl]-2-aminoethyl}glutamide) (C3-Car), and Poly(*N*-{*N′*-[*N″*-(4-carboxybutyl)-2-aminoethyl]-2-aminoethyl}glutamide) (C4-Car)

Side chains of PBLG were modified with DET-C*n*-Car (*n* = 2, 3, 4) *via* aminolysis (Scheme S4a). Briefly, PBLG (50 mg, 2.31 μmol, 225 μmol of the benzyl group in PBLG) and 2-hydroxypyridine (107 mg, 1.12 mmol, 5 eq. to the benzyl group in PBLG) were dissolved in 5 mL THF followed by the separate addition of DET-C2-Car *t*Bu (803 mg, 3.47 mmol, 15 eq. to the benzyl group in PBLG), DET-C3-Car *t*Bu (852 mg, 3.47 mmol, 15 eq. to the benzyl group in PBLG), and DET-C4-Car *t*Bu (901 mg, 3.47 mmol, 15 eq. to the benzyl group in PBLG) to synthesize C2-Car, C3-Car, and C4-Car, respectively. The mixtures were concentrated using a rotary evaporator followed by stirring for 1 day at r.t. Completion of the reactions was confirmed by the absence of the peak of benzyl protons (C*H*_2_-C_6_H_5_, δ = 5.02 ppm) on the side chains of PBLG in the ^1^H NMR spectra obtained using DMSO-*d*_6_ at 25 °C. Prior to dialysis (molecular weight cutoff (MWCO): 3.5 kDa) against deionized water, the reaction mixtures were acidified with 40 mL of 1 M HCl aq. and further stirred for 5 days at 40 °C. The dialyzed solutions were lyophilized to achieve C2-Car (52.2 mg, yield: 80.3%), C3-Car (52.7 mg, yield: 77.3%), and C4-Car (53.6 mg, yield: 75.4%) as slightly yellow powders. These three polymers were further examined by SEC [RI-detector; column: Superdex 200 increase 10/300 GL (GE Healthcare Life Science, Buckinghamshire, U.K.); and eluent: 10 mM HEPES buffer with 500 mM NaCl at pH = 7.4].

### 2.21 Preparations of Poly(*N*-{*N′*-[*N″*-(2-sulfethyl)-2-aminoethyl]-2-aminoethyl}glutamide) (C2-Sul), Poly(*N*-{*N′*-[*N″*-(3-sulfopropyl)-2-aminoethyl]-2-aminoethyl}glutamide) (C3-sul)), and Poly(*N*-{*N′*-[*N″*-(4-sulfobutyl)-2-aminoethyl]-2-aminoethyl}glutamide) (C4-Sul)

Side chains of PBLG were modified with DET-C*n*-Sul (*n* = 2, 3, and 4) *via* aminolysis (Scheme S4b). Briefly, PBLG (50 mg, 2.31 μmol, 225 μmol of the benzyl group in PBLG) and 2-hydroxypyridine (107 mg, 1.12 mmol, 5 eq. to the benzyl group in PBLG) were dissolved in 5 mL THF followed by the separate addition of DET-C2-Sul (1.33 g, 3.47 mmol, 15 eq. to the benzyl group in PBLG), DET-C3-Sul (1.38 g, 3.47 mmol, 15 eq. to the benzyl group in PBLG), and DET-C4-Sul (1.42 g, 3.47 mmol, 15 eq. to the benzyl group in PBLG) to fabricate C2-Sul, C3-Sul, and C4-Sul, respectively. These mixtures were concentrated using a rotary evaporator and then stirred for 1 day at r.t. Completion of the reactions was verified by the absence of the peak of benzyl protons (C*H*_2_-C_6_H_5_: δ = 5.02 ppm) on the side chains of PBLG in the ^1^H NMR spectra acquired using DMSO-*d*_6_ at 25 °C. Thereafter, the reaction mixtures were separately dissolved in 5 mL of 1 M HCl aq. (CH_3_OH/water = 4:1), and then, CH_3_OH was evaporated. The obtained aqueous solutions were poured into 49 mL of TFA (the final concentration of water was 2%) followed by stirring at r.t. for 3 days. Before dialysis (MWCO: 3.5 kDa) against deionized water, TFA was removed by evaporation. The dialyzed solutions were lyophilized to achieve C2-Sul (49.1 mg, yield: 70.7%), C3-Sul (53.1 mg, yield: 74.1%), and C4-Sul (51.3 mg, yield: 69.3%) as slightly yellow powders. These three polymers were further analyzed by SEC [RI-detector; column: Superdex 200 increase 10/300 GL (GE Healthcare Life Science, Buckinghamshire, UK); and eluent: 10 mM HEPES buffer with 500 mM NaCl at pH = 7.4].

### 2.22 Analysis of the Protonation Behaviors of Polymers by Potentiometric Titration

Prior to titration with 0.01 M NaOH containing 150 mM NaCl aq. using an automatic titrator (COM-1750, Hiranuma, Kyoto, Japan), the polymers (5 mg) were dissolved in 2 mL of 0.1 M HCl aq. comprising 150 mM NaCl aq. The titrant was added at 30 s intervals to stabilize the pH value. The relationship between the pH and the protonation degree of polymers (α) was evaluated from the obtained titration curves. Furthermore, p*K*_a_ values were calculated according to the Henderson–Hasselbalch equation (p*K* = pH + log[α/(1 – α)]), where *K* is the acid dissociation constant.

### 2.23 Fluorescence Labeling of the Polymers with Cy3

C*n*-Car and C*n*-Sul (*n* = 2, 3, and 4) were labeled with Cy3 *via* a Cu-free click reaction of the azide termini of the polymers and Cy3-DBCO. Briefly, 0.80 μmol of each polymer was dissolved in 2 mL of 10 mM Na_2_CO_3_ prior to the addition of 0.96 μmol of Cy3-DBCO (dissolved in DMSO at 10 mg/mL) followed by stirring for 24 h at r.t. in the dark. The resulting reaction mixture was purified by ultrafiltration using Amicon (MWCO: 3 kDa) at 4 °C and further purified using a PD-10 column with 1 M NaCl aq. Then, the filtrate was dialyzed (MWCO: 3.5 kDa) against deionized water for 1 day in the dark, followed by lyophilization for further use. MeO-PEG_20k_-NH_2_ was also labeled with Cy3. Precisely, 5.0 μmol of MeO-PEG_20k_-NH_2_ was dissolved in 2 mL of DCM, followed by the introduction of 6.0 μmol of Cy3-NHS ester (dissolved in DMSO at 5 mg/mL) and a catalytic amount of TEA. Thereafter, the resulting mixture was stirred for 24 h at room temperature in the dark followed by dialysis (MWCO: 3.5 kDa) against methanol and deionized water in the dark and lyophilization.

### 2.24 Fluorescence Labeling of MeO-PEG_20k_-NH_2_ with FITC

MeO-PEG_20k_-NH_2_ (5.0 μmol) was dissolved in 2 mL of acetone, followed by the addition of 6.0 μmol of FITC (dissolved in DMSO at 10 mg/mL) and stirring for 24 h at r.t. in the dark. The resulting reaction mixture was dialyzed (MWCO: 3.5 kDa) against methanol and deionized water in the dark, followed by lyophilization.

### 2.25 Analyses of the Interactions of Polymers with Heparin and Serum by Fluorescence Correlation Spectroscopy (FCS)

FCS was performed using an LSM 710 confocal scanning microscope (Carl Zeiss, Germany) equipped with a Confocor3 module and Zeiss Plan-Apochromat 40× (NA 1.2) water immersion objective. For the Cy3-polymers, a 488 nm Ar laser and a 575 nm filter were used for excitation and emission, respectively. To investigate pH-responsive interactions of polymers with heparin, Cy3-C*n*-Car and Cy3-C*n*-Sul (final concentration: 200 nM) were incubated at pH = 6.0–8.0 (10 mM HEPES or MES buffer containing 150 mM NaCl) with/without heparin (100 μg/mL) for 1 h. To analyze the interactions of polymers with serum, the Cy3-polymers (final concentration: 200 nM) were separately incubated with 5, 10, 20, and 40% FBS for 1 h at pH = 7.4 (10 mM HEPES buffer comprising 150 mM NaCl) before FCS.

### 2.26 Cytotoxicity Assay

Huh-7 cells were seeded on 96-well plates at densities of 10,000 cells per well and incubated overnight at 37 °C. Then, the medium was replaced with 100 μL buffer solutions: 10 mM HEPES buffer with 150 mM NaCl (aq) (pH = 7.4). These solutions also separately contained C*n*-Car and C*n*-Sul at concentrations of 0.004, 0.04, 0.4, 4, and 40 μM per well, and the cells were further incubated at 37 °C for 4 h. Next, 100 μL of working solution of the LDH assay (DOJINDO Laboratories, Kumamoto, Japan) was introduced, and the absorbance at 490 nm for each well was measured using a microplate reader (iMark, BIO-RAD, CA). Percentage of LDH activity in each well was calculated as the ratio of the obtained values to the values in the control wells treated with lysis buffer. The results represent the mean with the standard error of the mean (SEM) acquired from six samples.

### 2.27 Cellular Uptakes of Polymers

Huh-7 cells were seeded on 24-well plates at densities of 50,000 cells per well and incubated overnight at 37 °C in 0.5 mL of modified DMEM containing 10% FBS. Thereafter, the medium was replaced with 0.5 mL buffer solutions: 10 mM HEPES buffer with 150 mM NaCl aq. (pH = 7.4) or 10 mM MES buffer comprising 150 mM NaCl aq. (pH = 6.5). These solutions also separately contained Cy3-C*n*-Car and Cy3- C*n*-Sul (final concentration: 1 μM). The cells were further incubated for 4 h at 37 °C. After the cells were washed with PBS and trypsinization, the Cy3 fluorescence intensities of the obtained cells were measured by flow cytometry using Guava easyCyte 6-2L (Merck Millipore, Germany). The results represent the mean with the standard deviation acquired from three samples.

### 2.28 Evaluation of the Endosomal Escape Capabilities of Polymers

Huh-7 cells were seeded on 24-well plates at densities of 50,000 cells per well and incubated at 37 °C overnight in 0.5 mL of modified DMEM containing 10% FBS. Then, the medium was replaced with 0.5 mL buffer solutions: 10 mM MES buffer with 150 mM NaCl (pH = 6.5) and 10 μM FITC-PEG. These solutions also separately comprised MeO-PEG_20k_-NH_2_, C2-Car, C3-Sul (final concentration: 4 μM), and chloroquine (final concentration: 100 μM). The cells were further incubated at 37 °C for 3–48 h. After washing the cells with PBS and trypsinization, FITC fluorescence intensities of the acquired cells were measured by flow cytometry using Guava easyCyte 6–2L (Merck Millipore, Germany). The results represent the mean with standard deviation obtained from three samples.

To examine endosomal escapes of the polymers, Huh-7 cells were seeded on 24-well plates at densities of 10,000 cells per well and incubated at 37 °C overnight in 0.5 mL of modified DMEM containing 10% FBS. Thereafter, the medium was replaced with 0.5 mL of buffer solutions: 10 mM MES buffer with 150 mM NaCl aq. (pH = 6.5) and 100 μg of calcein. These solutions also separately comprised MeO-PEG_20k_-NH_2_, C2-Car, C3-Sul (final concentration: 10 μM), and chloroquine (final concentration: 500 μM). The cells were further incubated at 37 °C for 6 h. After the cells were washed with PBS, calcein fluorescence images were obtained using an all-in-one fluorescence microscope BZ-X710 (KEYENCE, Japan).

### 2.29 Computational Method

All calculations were performed using Gaussian 16 (Revision C.01).^27^ DFT calculations were conducted using the ω-B97X-D functional^28^ with tight self-consistent field convergence and ultrafine integration grids. The standard 6-311+G(d,p)^29^ basis set was used for all atoms. The solvent effect of water was considered using the polarizable continuum model (PCM)^30^ for DFT calculations. Each stationary point was adequately characterized by a normal coordinate analysis (no imaginary frequency for an equilibrium structure). All molecular graphics were prepared using CYLview20.^31^ Initial structures were generated by performing a conformer distribution search using Spartan’20.^32^ In all calculations, the temperature was set to 298.15 K. All optimized geometries are available in the Supporting Information in .xyz format.

### 2.30 Statistical Analysis

One-way analysis of variance (ANOVA) with Tukey’s multiple comparisons post hoc test or Student’s *t*-test was employed to assess statistical differences. Data analysis was conducted using GraphPad Prism version 7.00 for Windows (GraphPad Software). *p* < 0.05 was considered statistically significant for each analysis. For the statistical evaluation of time-dependent changes in fluorescence intensity (Figure 6), two-way ANOVA with Tukey’s multiple comparisons post hoc test was performed using GraphPad Software. All data were presented as means ± SEM.

## 3 Results

### 3.1 Synthesis and Characteristics of the Polyzwitterions

The combinations of anionic and cationic groups and C spacers are fundamental for understanding the roles of chemical structures in pH responsiveness. Thus, we designed and synthesized a series of EDA-based zwitterions on poly(l-glutamic acid) (C*n*-Car and C*n*-Sul, where *n* = 2, 3, and 4; Scheme S4 and Figures S2–7). PBLG was prepared by ring-opening polymerization of BLG-NCA using 1-azido-3,6,9-trioxaundecan-1-amine as the initiator (Scheme S3 and Figure S1). Gel permeation chromatography and ^1^H NMR spectroscopy revealed that the average degree of polymerization (DP) of the obtained PBLG was 97. Furthermore, the acquired polymer exhibited a relatively narrow polydispersity (*M*_w_/*M*_n_ = 1.18). Thereafter, the side chains of PBLG were subjected to aminolysis with zwitterionic precursors (Schemes S1 and S2), followed by the removal of the ester groups to obtain C*n*-Cars and C*n*-Suls. The structures of the acquired polyzwitterions were again confirmed by ^1^H NMR spectroscopy (Figures S2–S7).

### 3.2 Protonation Behaviors of the Polyzwitterions

**Scheme 1:**
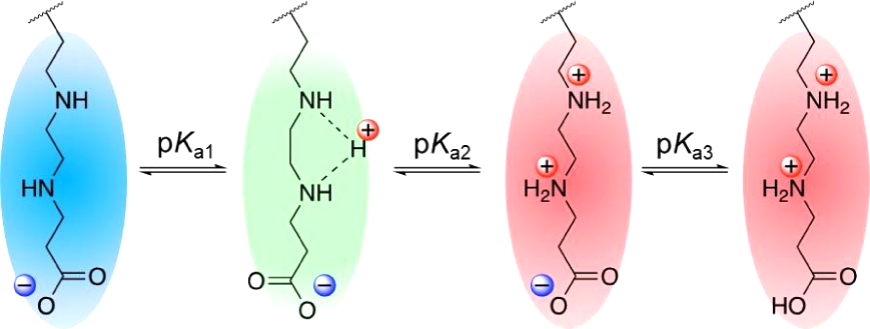
Stepwise Protonation Behaviors of the Polyzwitterions Synthesized in This Study Each equilibrium state is characterized by p*K*_a_.

To investigate the net charges of C*n*-Car and C*n*-Sul under physiological and tumor conditions (pH = 7.4 and 6.5), their protonation behaviors were evaluated *via* potentiometric titration in the pH range from 1.2 to 12.0. Physicochemical parameters, such as α, ratio of the cationic charge to anionic charge (*C*/*A*), and p*K*_a_ values, of the obtained polymers were estimated from α/pH and p*K*/α curves (Figures S8 and S9) and are presented in Table 1. *C*/*A* values of C*n*-Car and C*n*-Sul varied in the range from 1.09 to 1.25 at pH = 7.4 and then increased up to 1.53 at pH = 6.5, indicating that the net charges of C*n*-Car and C*n*-Sul changed from neutral to cationic. Additionally, the number of carbons in the spacer molecules influenced the *C*/*A* values; with an increase in the *C* length, the *C*/*A* value increased from 1.09 to 1.25 for C*n*-Car and from 1.06 to 1.14 for C*n*-Sul at pH = 7.4. Similar trends were observed at pH = 6.5. The EDA moieties in the polyzwitterions maintained two distinct p*K*_a_ values (p*K*_a1_ and p*K*_a2_). Note that p*K*_a1_ refers to the first protonation process, which produces a monovalent cation and renders the net charges of the polymers electrically neutral. In contrast, p*K*_a2_ refers to the second protonation process, which provides electrically cationic properties due to the formation of divalent cations on the polymer side chain (Scheme 1). Specifically, the fine-tuning of p*K*_a2_ is important because p*K*_a2_ directly affects the recognition of a slight difference in pH in the body, *i.e.*, the difference between neutral bloodstream and the acidic tumor microenvironment. Herein, the p*K*_a2_ values increased with an increase in the *C* spacer length, suggesting a net cationic charge in the polyzwitterion in a slightly acidic environment. p*K*_a3_ values of C*n*-Sul were not calculated because C*n*-Sul was not fully protonated, even at pH = 1.2.^33^

**Table 1:** Physicochemical Parameters of Polyzwitterions at pH = 7.4 and 6.5

	α (%)		*C*/*A*^a^				
polymer	pH = 7.4	pH = 6.5	pH = 7.4	pH = 6.5	p*K*_a1_	p*K*_a2_	p*K*_a3_
C2-Car	36.3	46.0	1.09	1.38	8.8	6.2	4.5
C3-Car	38.1	47.5	1.14	1.43	8.9	6.3	4.6
C4-Car	41.5	50.8	1.25	1.53	9.1	6.5	4.9
C2-Sul	35.4	44.3	1.06	1.33	8.7	6.1	-
C3-Sul	36.2	46.2	1.09	1.39	8.6	6.2	-
C4-Sul	37.8	46.8	1.14	1.41	8.8	6.4	-

a *C*/*A* is the ratio of the cationic charge to anionic charge.

### 3.3 Cationic Properties of Polyzwitterions

**Figure 2:**
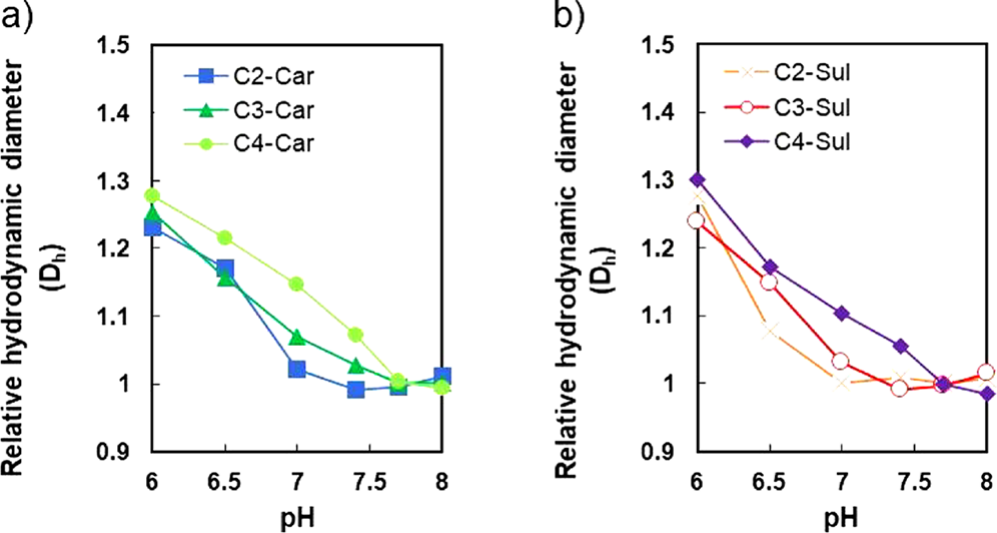
Relative hydrodynamic diameters of Cy3-labeled (a) C*n*-Car and (b) C*n*-Sul after 1 h incubation with heparin at pH = 6.0–8.0.

The goal of this study was to design and develop net-charge-controllable polymers that can recognize a narrow pH window in the body by altering their side chain structures in response to pH changes. These polymers can be used as structural components of systemically injectable materials/nanomedicines. Thus, their ability to interact with cells is an important factor. At first, we evaluated the interactions between the polymers fabricated herein and in heparin by measuring the change in hydrodynamic diameter (*D*_h_) with respect to pH in the range of 6.0–8.0 using FCS. Because heparin is an anionic component of the glycocalyx, polymer–heparin complex formation and the subsequent increase in *D*_h_s can be detected when an electrostatic interaction is induced by the cationic property of C*n*-Car or C*n*-Sul. Both Cy3-labeled C*n*-Car and C*n*-Sul were prepared *via* a click reaction using Cy3-DBCO before FCS. The FCS results and relative *D*_h_s of the series of Cy3-polyzwitterions are depicted in Figure 2. The relative *D*_h_s of C*n*-Car and C*n*-Sul increased with a decrease in pH. Considering the stability in the bloodstream, lower electrostatic interactions are preferable at pH = 7.0–7.4. In this regard, C3-Car and C4-polymers demonstrated high *D*_h_s owing to the formation of their complexes with heparin in this pH range. On the contrary, C2-polymers and C3-Sul were stable in the presence of heparin at pH = 7.0–7.4. Additionally, their relative *D*_h_s started to increase when the pH was less than 7.0, indicating the potential for acidic pH-responsive interactions between these polymers (C2-Car, C2-Sul, and C3-Sul) and heparin.

**Figure 3:**
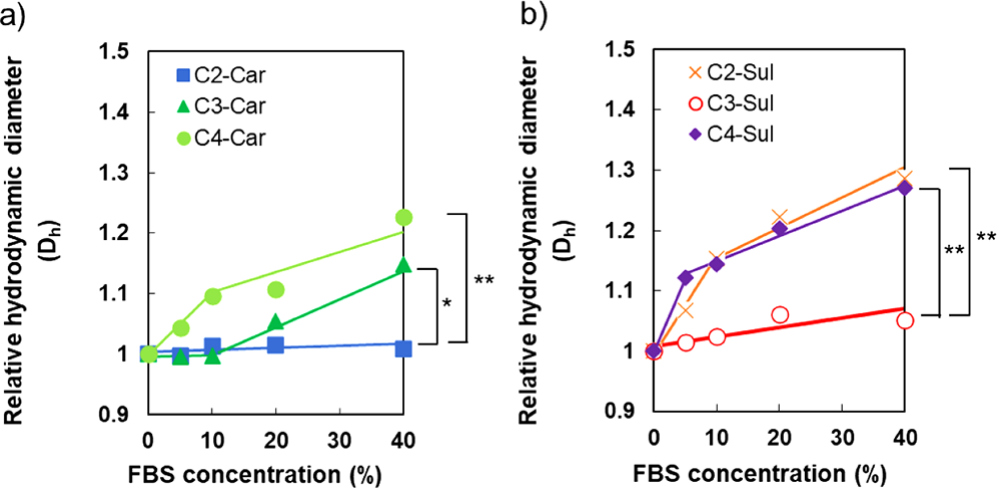
Relative hydrodynamic diameters of Cy3-labeled (a) C*n*-Car and (b) C*n*-Sul after 1 h incubation with FBS at pH = 7.4. Data are shown as mean ± SEM, *n* = 10, *p < 0.05 and **p < 0.01 (one-way ANOVA with Tukey’s multiple comparisons test).

Subsequently, we confirmed the antifouling properties of C*n*-Car and C*n*-Sul by measuring *D*_h_ at pH = 7.4 in the presence of serum (FBS), which is a major fluid component of blood containing several proteins (Figure 3). Sizes of C2-Car and C3-Sul exhibited fewer variations at all of the concentrations tested. As expected from the *C*/*A* values (Table 1), *D*_h_s of C4-polymers demonstrated concentration-dependent increments, suggesting strong interactions between these polymers (C4-Car and C4-Sul) and FBS. C3-Car exhibited a weaker interaction with FBS than that of C4-polymers. Interestingly, C2-Sul also demonstrated the same concentration-dependent increasing tendency.

### 3.4 Membrane Destabilizing Effects of Polyzwitterions for Cultured Cells

**Figure 4:**
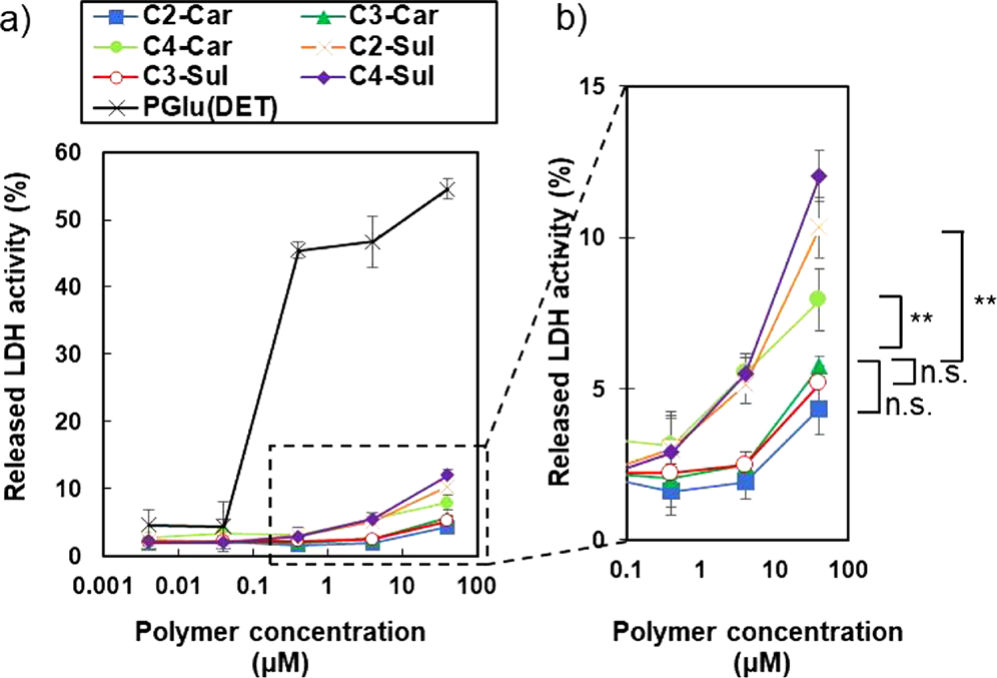
Activity of lactase dehydrogenase (LDH) released from cultured Huh-7 cells after separate treatments with C*n*-Car, C*n*-Sul, and PGlu(DET) homopolymer for 4 h at pH = 7.4. (a) Overall profiles of LDH activity. (b) Expanded profiles. Data are shown as mean ± SEM, *n* = 6, **p < 0.01 (one-way ANOVA with Tukey’s multiple comparisons test).

To examine the membrane destabilizing effects of C*n*-Car and C*n*-Sul, Huh-7 cells were separately incubated with C*n*-Car and C*n*-Sul at various concentrations (from 0.004 to 40 μM) and pH = 7.4. The LDH activity was analyzed. We used poly (glutamic acid)-based cationic homopolymer (PGlu(DET)) as a positive control polymer because this polymer comprises naked EDA groups on its side chain, making the polymer strongly positively charged at pH = 7.4. Figure 4 shows the percentage of LDH activity for each polymer. LDH activities of all polyzwitterions were substantially lower than that of PGlu(DET) homopolymer because the insertion of negatively charged groups into the ends of the side chains neutralized the cationic charges of the EDA moiety. Particularly, significantly less LDH activities (<5%) were observed for C2-Car, C3-Car, and C3-Sul, even at 40 μM, as compared to those of the other polymers. The lower membrane destabilizing effects of the polyzwitterions can be explained by their negligible interactions with cell membrane components under physiological conditions.

### 3.5 Cellular Uptakes of Polyzwitterions

**Figure 5:**
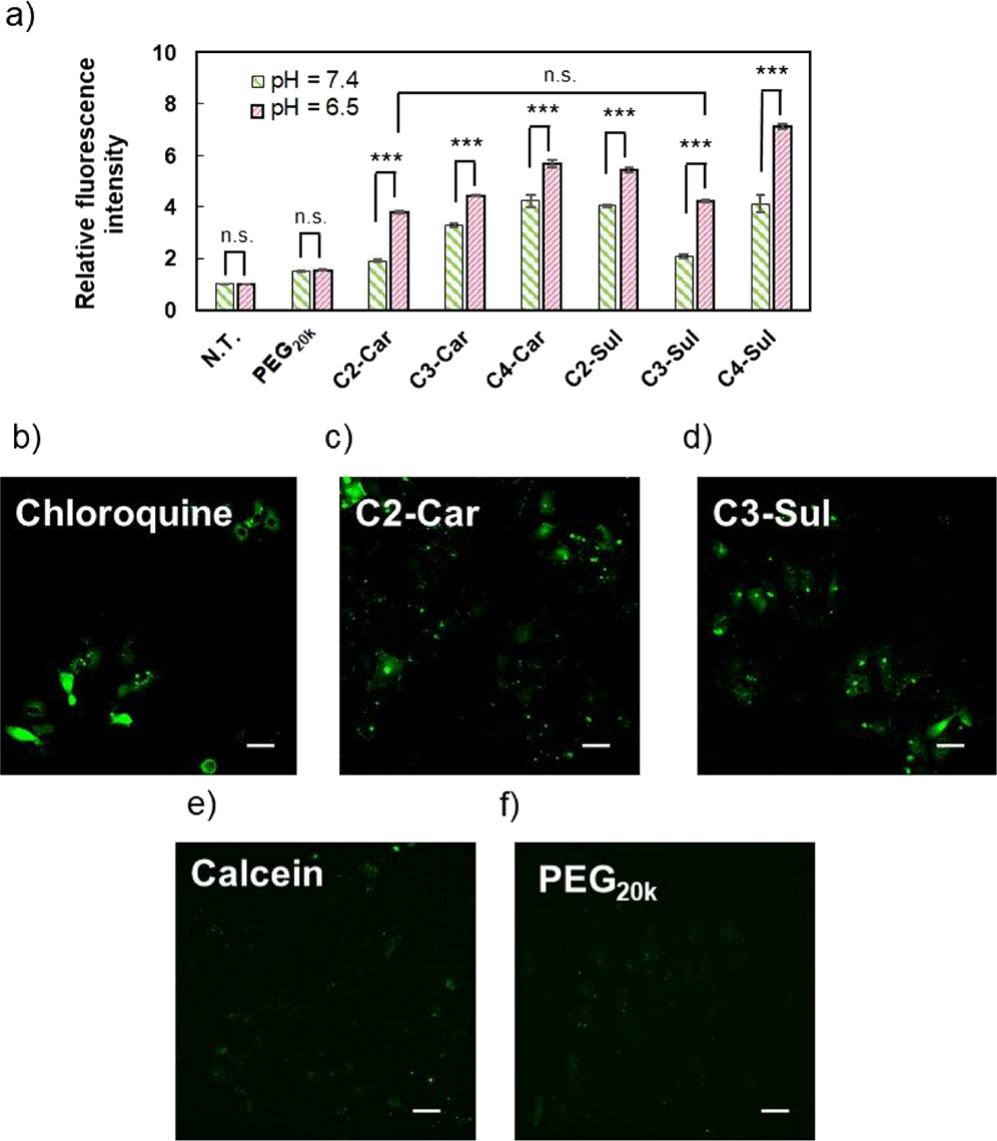
(a) Cellular uptakes of Cy3-labeled polyzwitterions by cultured Huh-7 cells after 4 h treatments at pH = 7.4 and 6.5. Data are shown as mean ± SEM, *n* = 3, ***p < 0.001 (Student’s *t*-test). (b)–(f) Intracellular distributions of calcein for cultured Huh-7 cells after separate treatments with calcein, polymers, and chloroquine at pH = 6.5. Scale bars: 50 μm.

Huh-7 cells were separately coincubated with Cy3-labeled C*n*-Car and C*n*-Sul at both pH = 7.4 and 6.5. Flow cytometry analysis revealed higher fluorescence signals of C*n*-Car and C*n*-Sul at pH = 6.5 than at pH = 7.4 (Figure 5a). Moreover, the control antifouling polymer PEG_20k_ did not demonstrate such a pH responsiveness. The fluorescence signals acquired for C2-Car and C3-Sul were comparable to that of PEG_20k_ at pH = 7.4, whereas they were significantly higher than that of PEG_20k_ at pH = 6.5, suggesting their higher cellular uptakes under the acidic tumor condition. Considering the utilization of the antifouling property in the body, C2-Car and C3-Sul should maintain equipotential abilities of cell interaction with PEG at pH = 7.4. Thus, we selected C2-Car and C3-Sul for further evaluation of intracellular behaviors, including endosomal escape abilities.

### 3.6 Endosomal Escape Abilities of Polyzwitterions

We used the calcein assay to evaluate the endosomal escape abilities of C2-Car and C3-Sul.^34,35^ Calcein is a membrane-impermeable fluorescent dye that can be cointernalized into cells by endocytosis with polymers. Furthermore, when endosomes are punctured due to the membrane destabilizing effect, calcein spreads into the cytosol and its fluorescence signal can be observed in the cytosol. As shown in Figure 5b–f, C2-Car and C3-Sul exhibited diffused fluorescence signals in the cytosol (Figure 5c,d), which were similar to that of the positive control group, *i.e.*, the chloroquine-treated group (Figure 5b).^36,37^ In contrast, the cells incubated with calcein alone (Figure 5e) or those treated with PEG_20k_ (Figure 5f) did not demonstrate these signals in the cytosol, indicating a lack of endosomal escape.

**Figure 6:**
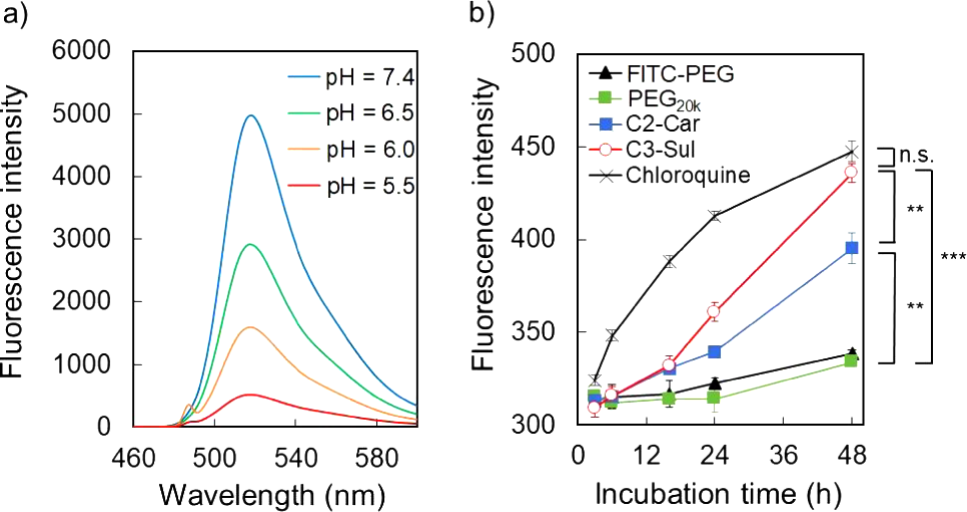
(a) Fluorescence emission spectra of 10 mM FITC-PEG at pH in the range from 5.5 to 7.4. (b) Time-dependent changes in the fluorescence intensity of fluorescein for Huh-7 cells separately cultured in chloroquine, C2-Car, C3-Sul, PEG_20k_, and FITC-PEG systems at pH = 6.5. Data are shown as mean ± SEM, *n* = 3, **p < 0.01 and ***p < 0.001 (two-way ANOVA with Tukey’s multiple comparisons test).

Although calcein is a common indicator for estimating endosomal escape, it is a small molecule with a molecular weight of 622.55 g/mol. During the analysis of endosomal behavior, the effect of molecular weight on endosomal behavior is inevitable and needs careful consideration. Therefore, for endosomal escape evaluation, the use of high-molecular-weight indicators is preferred to detect the endosomal escape profiles more precisely. We developed a pH-dependent fluorescence-quenching polymer to evaluate the endosomal escape abilities of C2-Car and C3-Sul. Fluorescence signal of the fluorescein-PEG conjugate FITC-PEG (*M*_n_ = ca. 20,000 g/mol) in this study exhibited pH-responsive fluctuation ability (Figure 6a), quenching under late endosome/lysosome conditions (pH = 5.5), and the signals become visible when FITC-PEG is released from the endosome to the cytosol (pH = 7.4). This FITC-PEG-based method enables quantification of the fluorescence signals only from the cytosol. Time-dependent endosomal escape changes in the fluorescence intensity of fluorescein for Huh-7 cells separately cultured in chloroquine, C2-Car, C3-Sul, PEG_20k_, and FITC-PEG systems at pH = 6.5 are depicted in Figure 6b. The fluorescence signal of positive control chloroquine rapidly increased from the initial stage of incubation. Fluorescence signals of the C2-Car- and C3-Sul-treated groups gradually increased over time, whereas those of the PEG_20k_ and FITC-PEG treatment groups negligibly increased. Moreover, an increase in LDH activities at pH = 5.5 was verified for both C2-Car and C3-Sul, indicating their pH-dependent membrane destabilization effects (Figure S10).

### 3.7 Conformational Stabilities of the Zwitterionic Moieties

**Figure 7:**
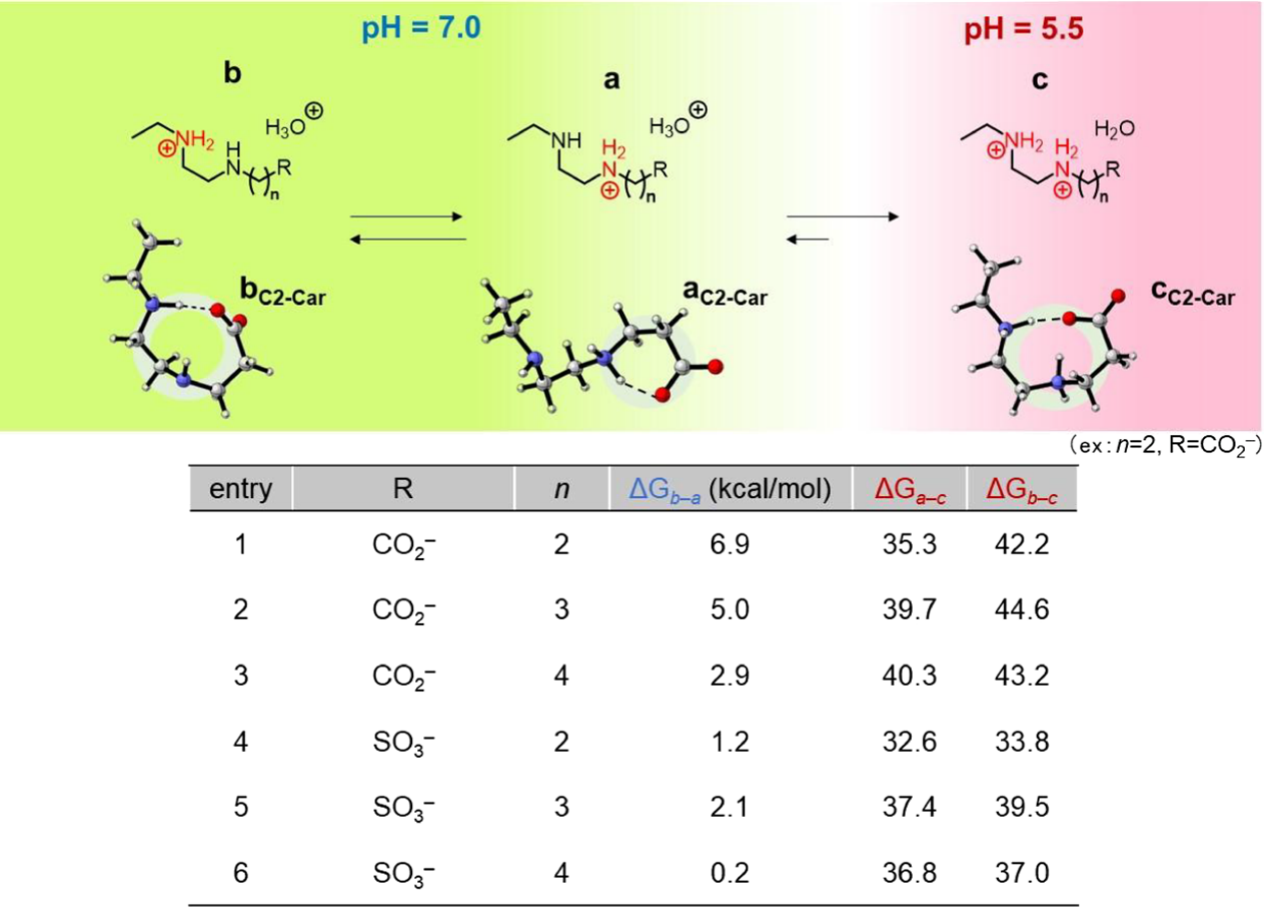
Conformational equilibria of the ethylenediamine-based zwitterionic moieties and free energy changes among the conformations.

We speculated that the side-chain conformation and intramolecular electrostatic association in the zwitterionic moieties were the key factors responsible for the above-mentioned unique properties of polyzwitterions; thus, we performed a conformational study *via* DFT calculations. A series of EDA-based zwitterionic moieties were designed *in silico*, and Gibbs free energy changes (*ΔG*s) were estimated. Potential chemical structures, conformations under different pH conditions, and *ΔG*s are shown in Figure 7. At neutral pH (7.0), zwitterionic moieties in equilibrium adopted two different conformations (**a** and **b**). The calculation results revealed that the conformational free energy changes from **a** to **b** (*ΔG*_*b*–*a*_) for all zwitterions exhibited positive values, suggesting that **a** is more stable than **b** and these equilibria are favorable toward **a** at neutral pH. Note that the anionic groups in the EDA-based zwitterionic moieties form associations with the neighboring amino groups for **a**, whereas they develop associations with the secondary neighboring amino groups for **b** and **c**. C2-Car and C3-Sul systems afforded relatively higher *ΔG*_*b*–*a*_ values among C*n*-Car and C*n*-Sul, respectively, which were in accordance with the better antifouling properties of C2-Car and C3-Sul in the current evaluations. Furthermore, the two amino groups were fully protonated at pH = 5.5, thereby forming **c**. Compared to *ΔG*_*b–a*_, *ΔG_a–c_* and *ΔG*_*b–c*_ were considerably larger, suggesting that **c** became significantly more stable than **a** and **b** with a decrease in pH, and thus, **c** was the dominant conformation under acidic conditions.

## 4 Discussion

Polymers play critical roles in drug formulations.^1,38,39^ Among them, polyzwitterions are well-known materials/pharmaceutical additives that show unique properties such as antifouling property. Nevertheless, the detailed relationship between the chemical structures of the zwitterionic moieties on the polymer and their biological performance remains to be fully clarified. Thus, herein, we synthesized a new series of EDA-based polyzwitterions and studied their characteristics. Additionally, the potential interaction between the obtained polyzwitterions and biomolecules was investigated for future applications with nanomedicines.

For the exhibition of antifouling property during blood circulation, the net charge of polyzwitterions is a crucial factor. We have used the *C*/*A* value as an indicator of the charge status, and we previously identified that the *C*/*A* value of polyzwitterions needed to be less than 1.10 to maintain the antifouling property under physiological condition. Indeed, our EDA-based polyzwitterions, *i.e.*, C2-Car, C2-Sul, and C3-Sul polymers with *C*/*A* values less than 1.10 (pH = 7.4), did not interact with heparin (Figure 2). The formation of aggregates was confirmed in the mixture of heparin and C3-Car/C4-Sul, which was equipped with a *C*/*A* value of 1.14 (pH = 7.4), indicating that the *C*/*A* value of 1.10 is a potential boundary condition for the expression of antifouling. Further experiments were performed by evaluating interaction with FBS, and those results presented superior antifouling properties in C2-Car and C3-Sul (Figure 3). Given the preferable antifouling of C2-Car/C3-Sul compared with that of C2-Sul, there is another factor that connects the antifouling function and chemical structures. Although some previous reports have provided physicochemical evidence regarding the effect of C spacer length on counterions and molecular hydrations,^25,40,41^ little attention has been paid to the steric conformation of each zwitterionic side chain and its related stability. Therefore, in this study, we focused on the relationship between the C spacer lengths and the steric conformations. C2-Car and C3-Sul exhibited adequately high *ΔG*_b–a_ values (Figure 7), implying their most stable conformations at pH = 7.4. Hence, the stability of the conformation in the zwitterion structure is one of the important factors to achieve a favorable performance of antifouling.

It has been known that intratumoral pH decreases to 6.5–7.0 by the Warburg effect,^16−19^ and our C2-Car/C3-Sul polymers showed the preferable alternation of net charge and antifouling property in this narrow pH range (Figures 2, 5, and 6). Although various polyzwitterions have been reported, our polymer was specially designed to recognize such a narrow pH window, maximizing the selective cellular uptake of C2-Car/C3-Sul-coated nanomedicines in the tumor microenvironment. Additionally, as shown in Figures 5 and 6, the pH-responsiveness of C2-Car/C3-Sul polymers could also contribute to the resolution of the PEG dilemma, a common challenge in drug delivery of nucleic acids therapeutics. Previously, we synthesized other pH-responsive polymers, *i.e.*, poly{*N*-[*N*′-(2-aminoethyl)-2-aminoethyl]aspartamide} (PAsp(DET)) and PGlu(DET) that showed the high efficiency of endosomal escape due to the diprotonation effects under acidic conditions, and they showed the practicability for drug delivery of nucleic acids such as plasmid DNA and mRNA.^14,15^ The pH-responsiveness of EDA-based polyzwitterions was driven by similar diprotonation effects but with superior sensitivity against the narrow tumorous pH window. Another point to note is the effect of DP on the characteristics of polyzwitterion-coated nanomedicines. We have investigated the influence of the DP in similar but different ethylenediamine-based poly(carboxybetaine)-lipid conjugates and those lipid nanoparticles (LNPs).^42^ Then, we found that the obtained LNPs exhibited enhanced cellular uptakes with increasing DP values at pH 6.5. However, the polyzwitterion with a higher DP value was revealed to disrupt the membrane fusion by LNP, resulting in reduced siRNA transfection. Moreover, all LNPs, regardless of DP values, demonstrated negligible cytotoxicity against cancer cell. Thus, although the polyzwitterions with different DP showed similar characteristics, the obtained nanostructured particles, e.g., polymeric micelle, LNP, and liposome, have a potential to show different functional properties depending on their DPs. Hence, further research is needed to reveal more details, and we are currently studying such properties of C2-Car/C3-Sul-coated nanomedicines.

## 5 Conclusions

In summary, we developed a series of EDA-based polyzwitterions and found the importance of *C*/*A* value and relationship between anionic groups and *C* spacer lengths for exhibiting antifouling property. DFT calculations revealed the principle of conformation stability, *i.e.*, *ΔG*s, for tuning the sensitivity of antifouling in a narrow pH window. Additionally, our EDA-based polyzwitterions, especially C2-Car/C3-Sul polymers, showed the amplification of cellular uptake and endosomal escape in response to tumoral acidic environment. Overall, the present study highlights the importance of designing the polymer and its function for antifouling and biointeractive properties in response to the surrounding environment and thus contributes to the development of future smart nanomedicines.

## Supporting Information Available

Scheme of the synthesis of DET-C*n*-Car *t*Bu, DET-C*n*-Sul TFPT, PBLG, C*n*-Car, and C*n*-Sul; ^1^H NMR spectra of PBLG, C2-Car, C3-Car, C4-Car, C2-Sul, C3-Sul, and C4-Sul in D_2_O; α/pH curves of C*n*-Car and C*n*-Sul; p*K*/α curves of C*n*-Car and C*n*-Sul; activity of lactase dehydrogenase; computational parameters (PDF)
