# Supplementary material for: Synthesis and Optimization of Ethylenediamine-Based Zwitterion on Polymer Side Chain for Recognizing Narrow Tumorous pH Windows
Source: Biomacromolecules. 2024 Oct 31;25(12):7788–98. doi: 10.1021/acs.biomac.4c01086 (PMC11632656; doi:10.1021/acs.biomac.4c01086)
Supplement: Supplementary file 1 — bm4c01086_si_001.pdf [file bm4c01086_si_001.pdf]

Supporting Information

Synthesis and Optimization of Ethylenediamine-Based Zwitterion on Polymer Side Chain for Recognizing Narrow Tumorous pH Windows

Masahiro Toyoda,[†,‡] Yutaka Miura, [†,‡,§,*] Motoaki Kobayashi,[†] Masato Tsuda,[†,‡] Takahiro Nomoto,[◊] Yuto Honda,[†,§] Hiroyuki Nakamura,[†,‡] Hiroyasu Takemoto,[Δ] Nobuhiro Nishiyama[†,‡,§,*]

[†] Laboratory for Chemistry and Life Science, Institute of Innovative Research, Tokyo Institute of Technology, 4259 Nagatsutacho, Midori-ku, Yokohama, Kanagawa, 226-8501, Japan.

[‡] Department of Life Science and Technology, School of Life Science and Technology, Tokyo Institute of Technology, 4259 Nagatsutacho, Midori-ku, Yokohama, Kanagawa, 226-8501, Japan.

[§] Innovation Center of Nanomedicine (iCONM), Kawasaki Institute of Industrial Promotion, 3-25-14 Tonomachi, Kawasaki-ku, Kawasaki, Kanagawa, 210-0821, Japan.

[◊] Department of Life Sciences, Graduate School of Arts and Sciences, the University of Tokyo, Tokyo, 3-8-1 Komaba, Meguro-ku, Tokyo, 153-8902, Japan.

[Δ] Medical Chemistry, Graduate School of Medical Science, Kyoto Prefectural University of Medicine, 1-5 Shimogamohangi-cho, Sakyo-ku, Kyoto, 606-0823, Japan.

[*]Correspondence and requests for materials should be addressed to Y.M. (email: miura.y.ai@m.titech.ac.jp) and N.N. (email: nishiyama.n.ad@m.titech.ac.jp)

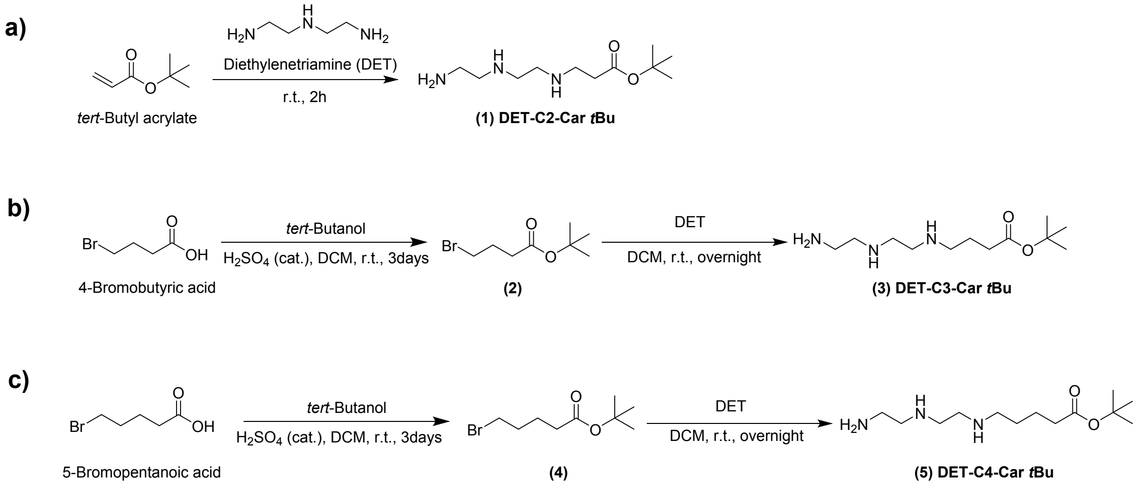

Scheme S1. Synthetic procedure of DET-C*n*-Car *t*Bu.

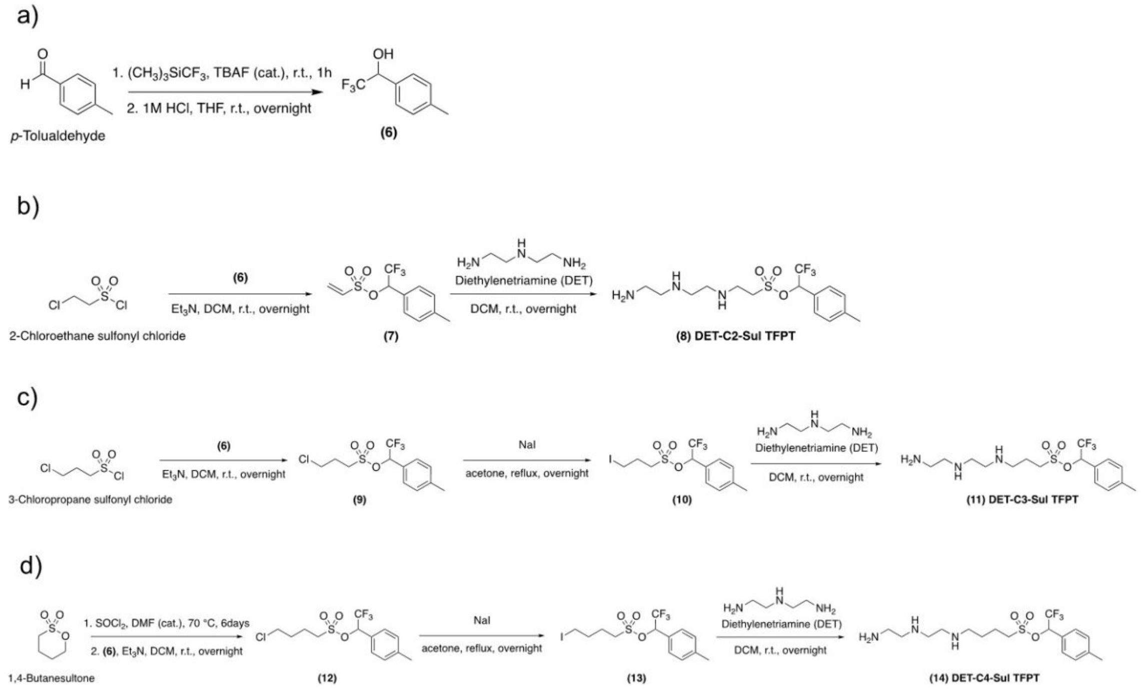

Scheme S2. Synthetic procedure of DET-C*n*-Sul TFPT.

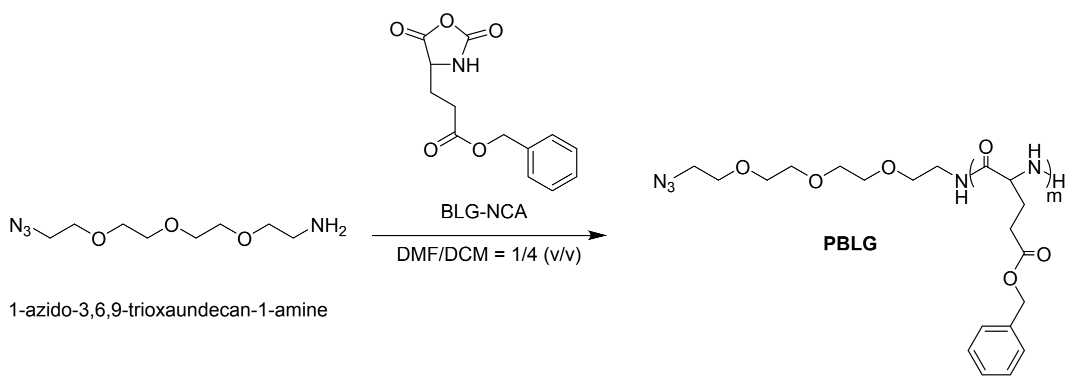

Scheme S3. Synthetic procedure of PBLG.

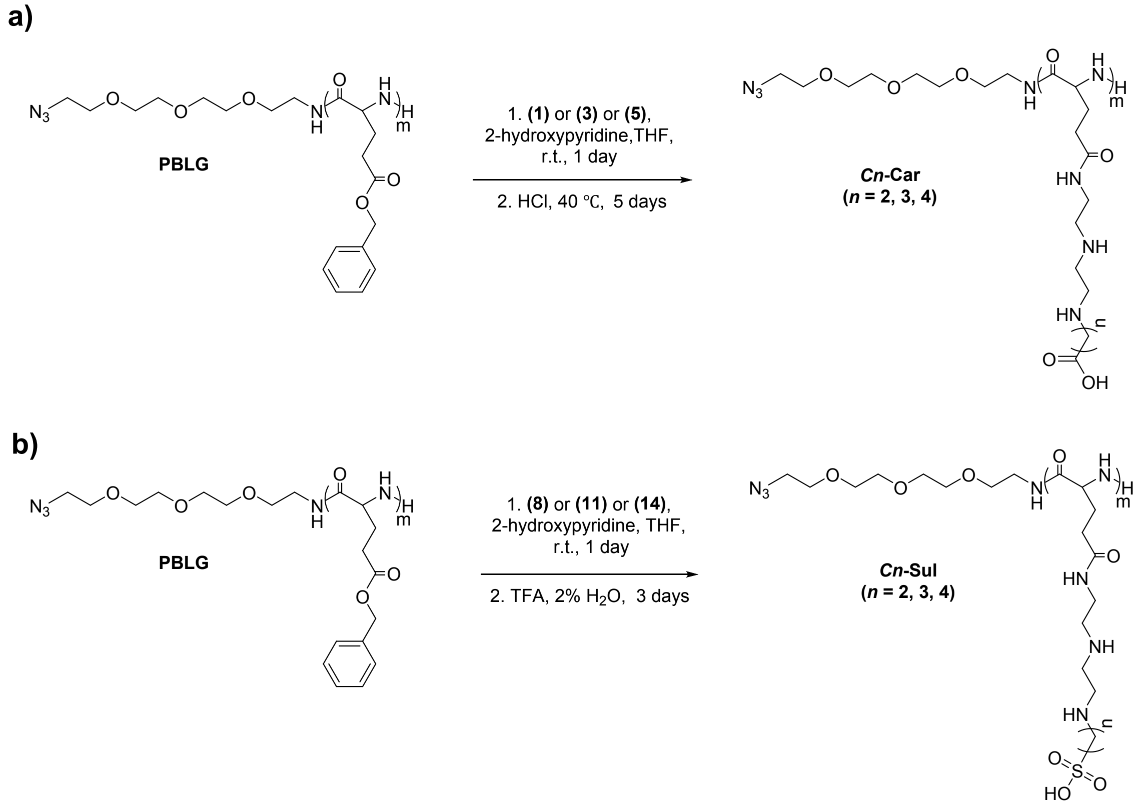

Scheme S4. Synthetic procedure of a) C*n*-Car and b) C*n*-Sul.

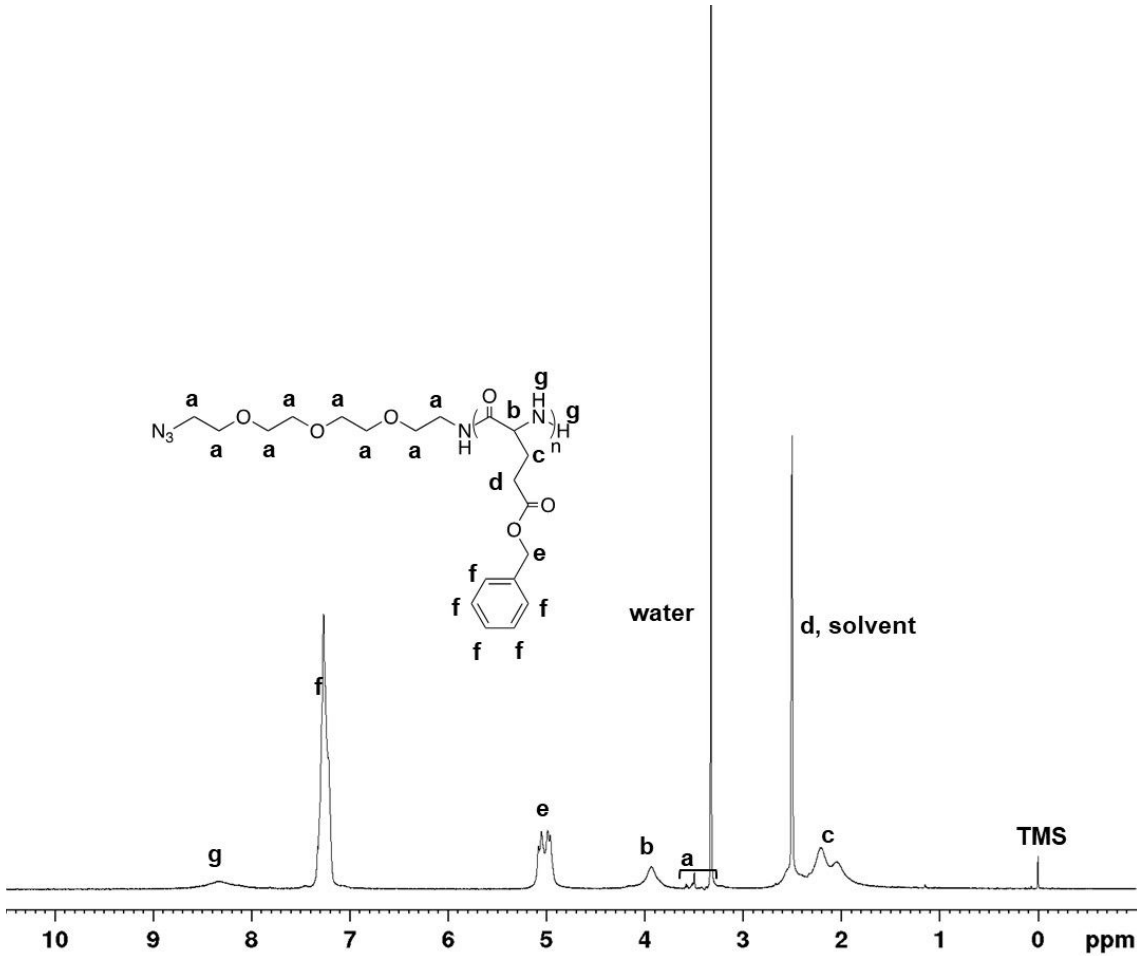

Figure S1. ^{1}H NMR spectrum of PBLG in DMSO-d_6.

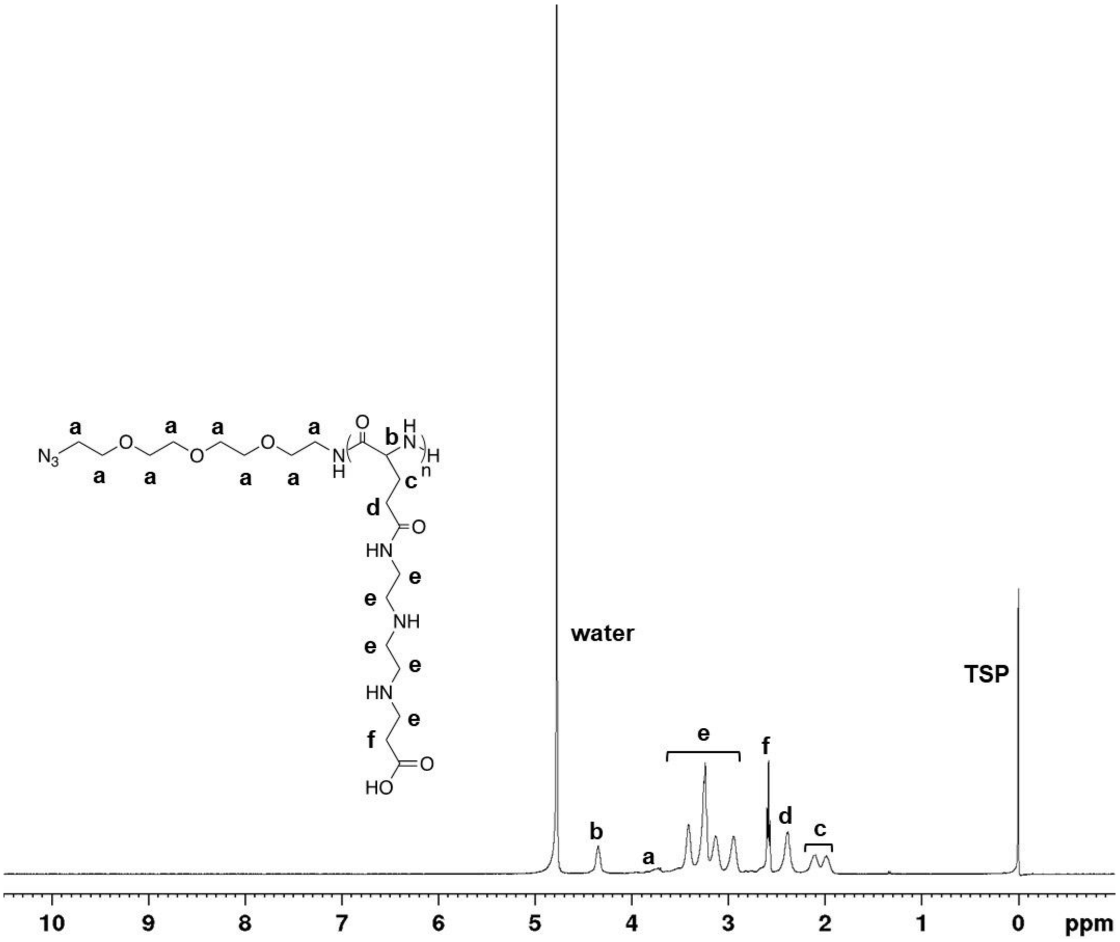

Figure S2. ^{1}H NMR spectrum of C2-Car in D_2O.

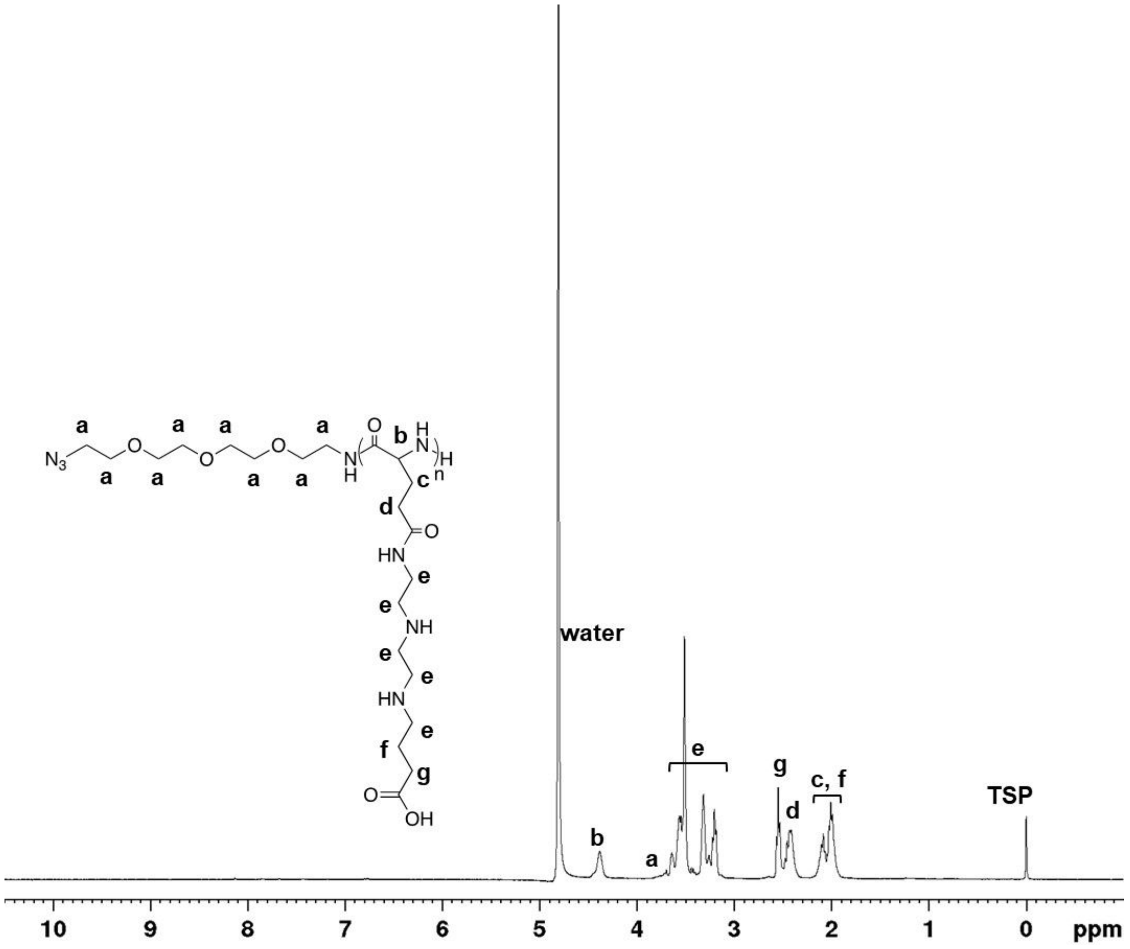

Figure S3. ^{1}H NMR spectrum of C3-Car in D_2O.

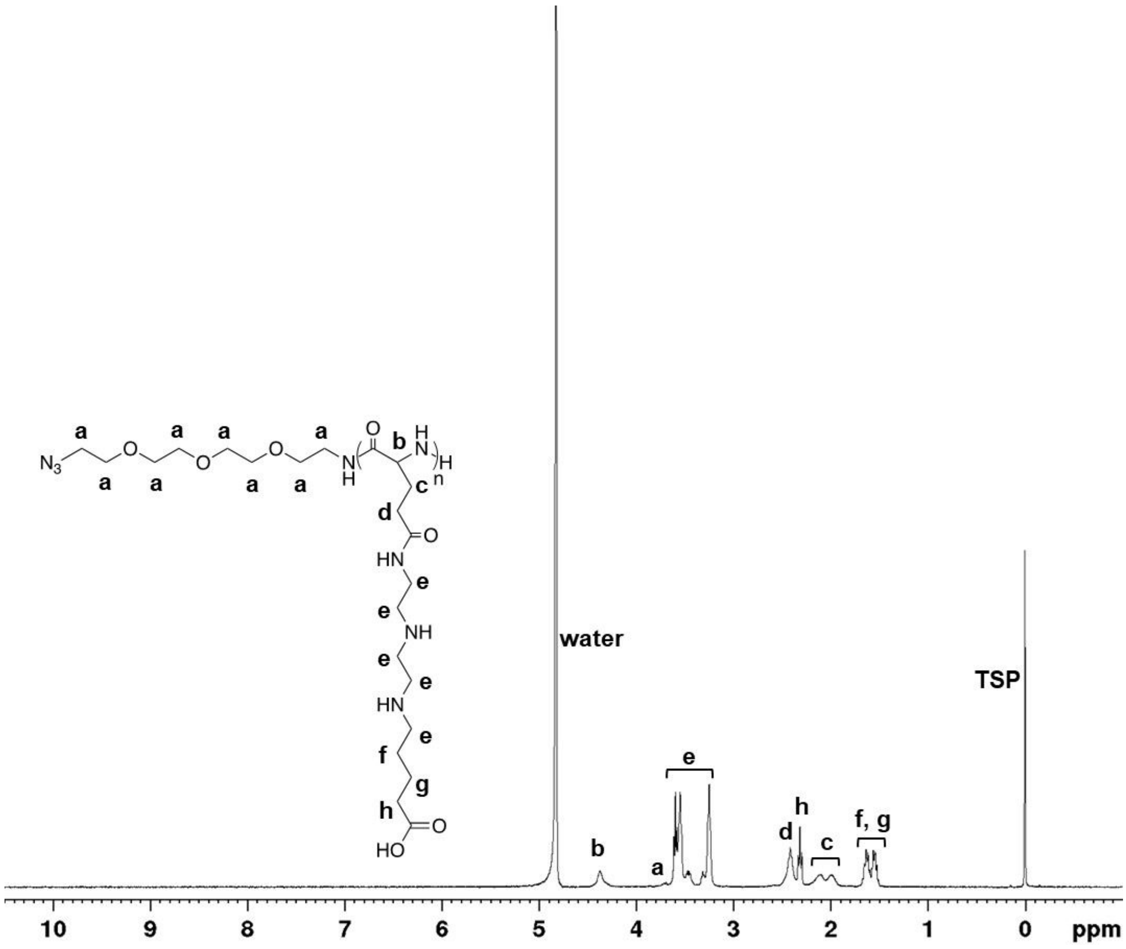

Figure S4. ^{1}H NMR spectrum of C4-Car in D_2O.

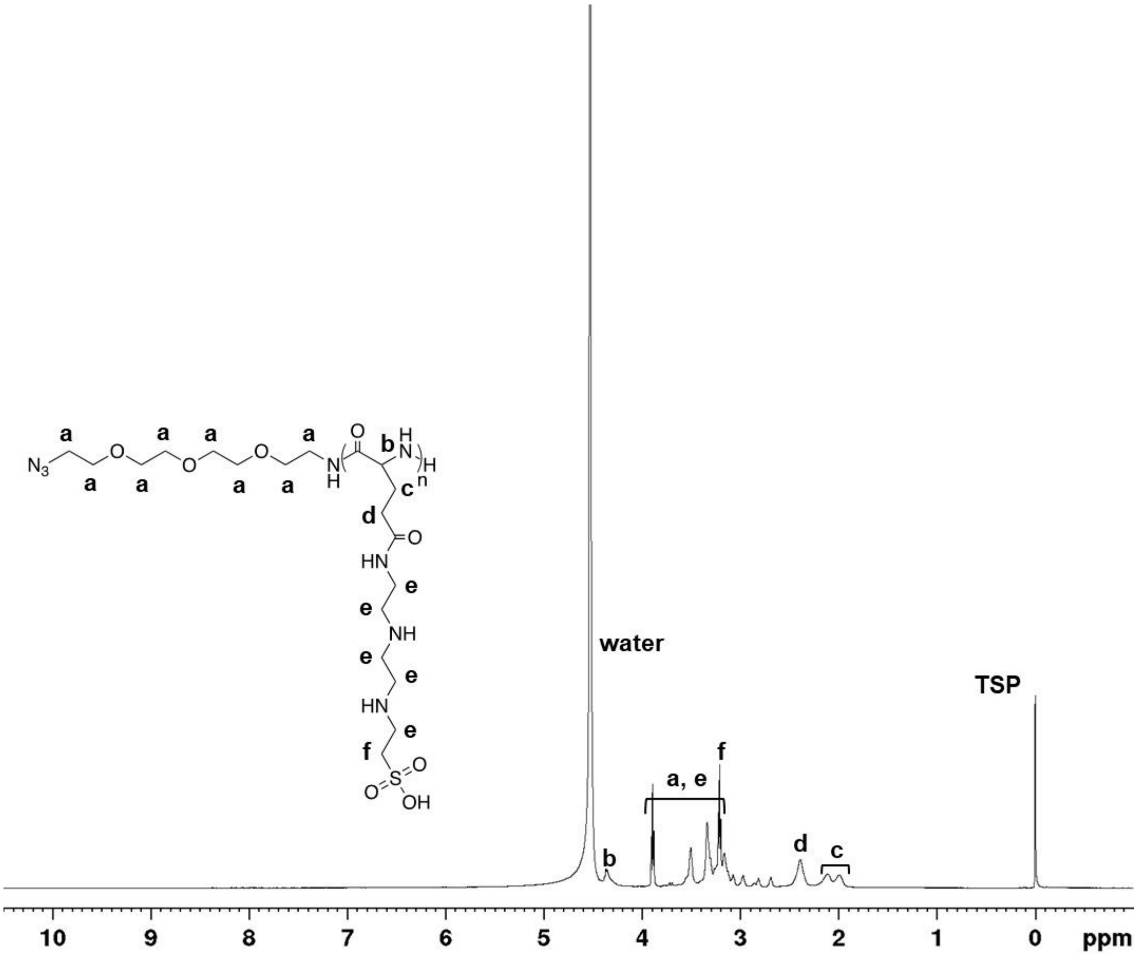

Figure S5. ^1H NMR spectrum of C2-Sul in D_2O.

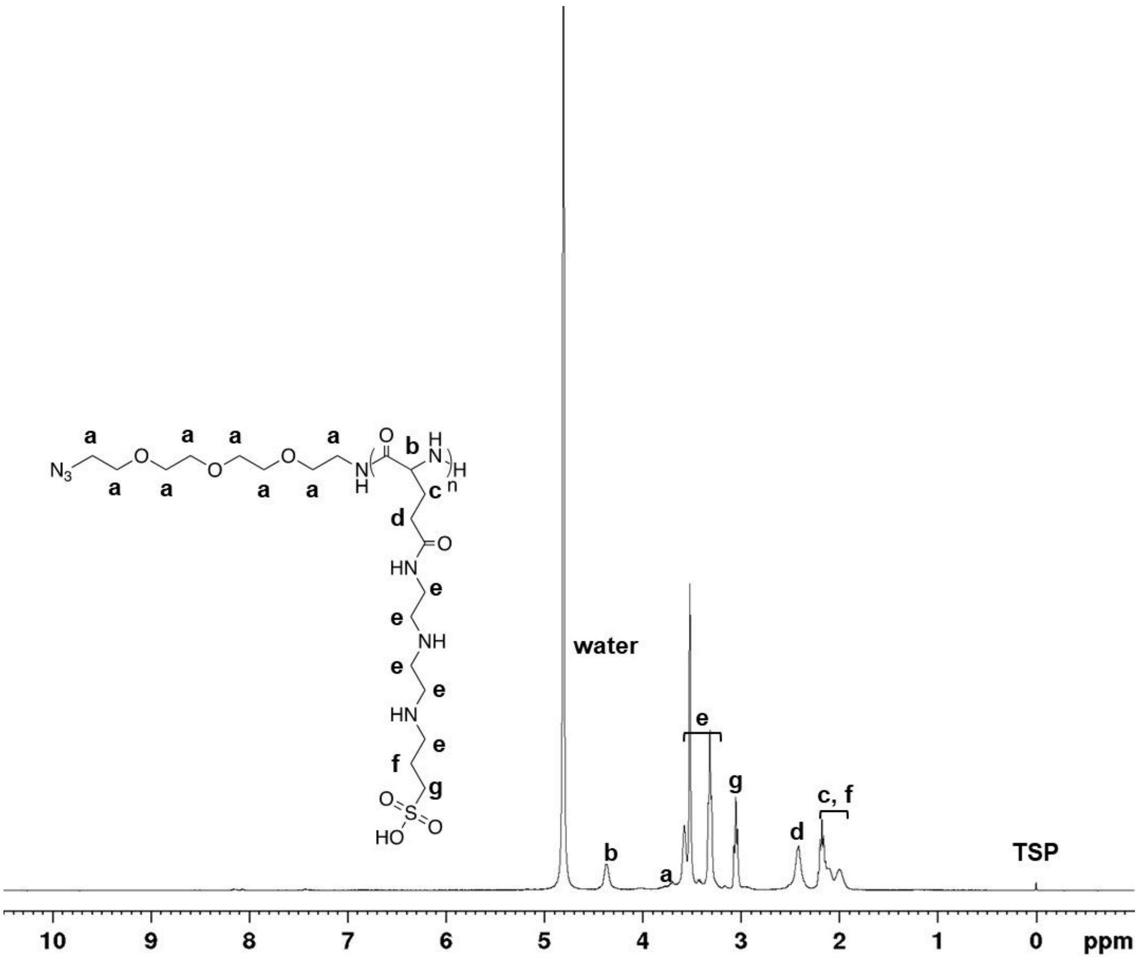

Figure S6. ^{1}H NMR spectrum of C3-Sul in D_2O.

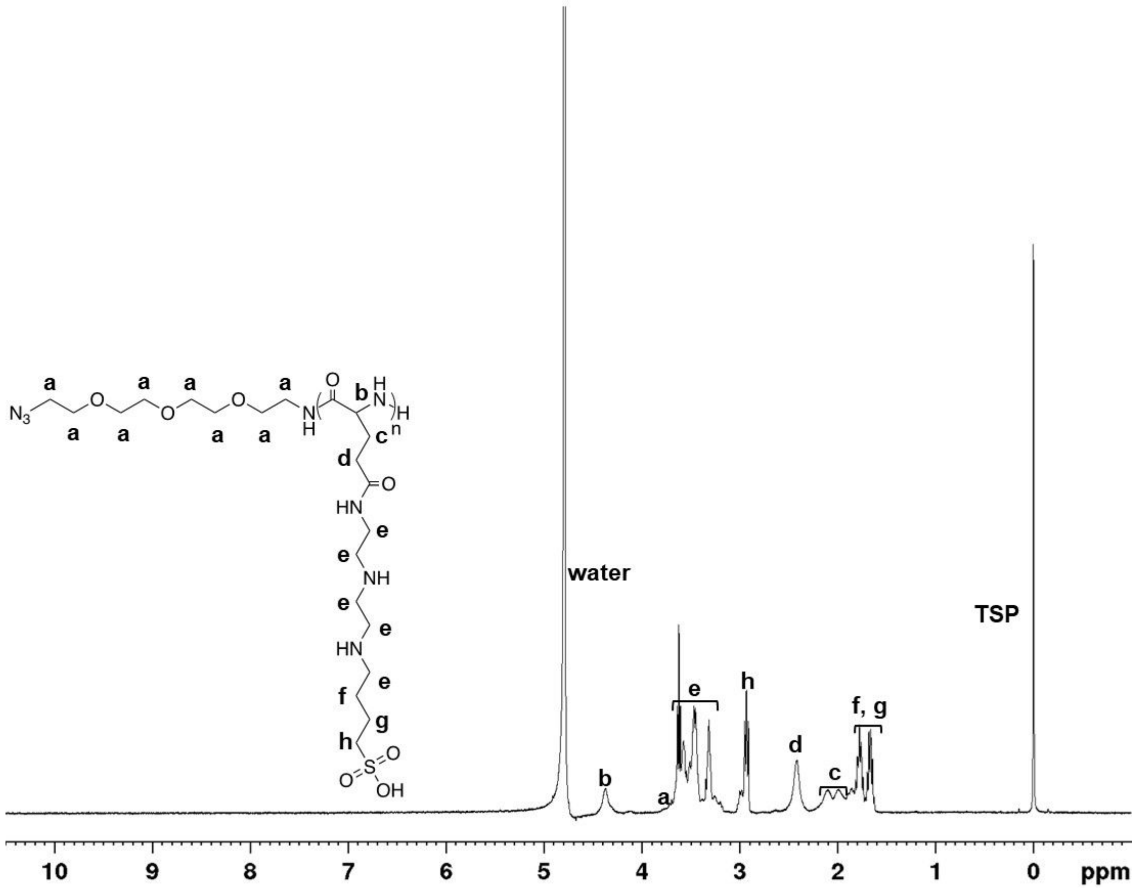

Figure S7. ^1H NMR spectrum of C4-Sul in D_2O.

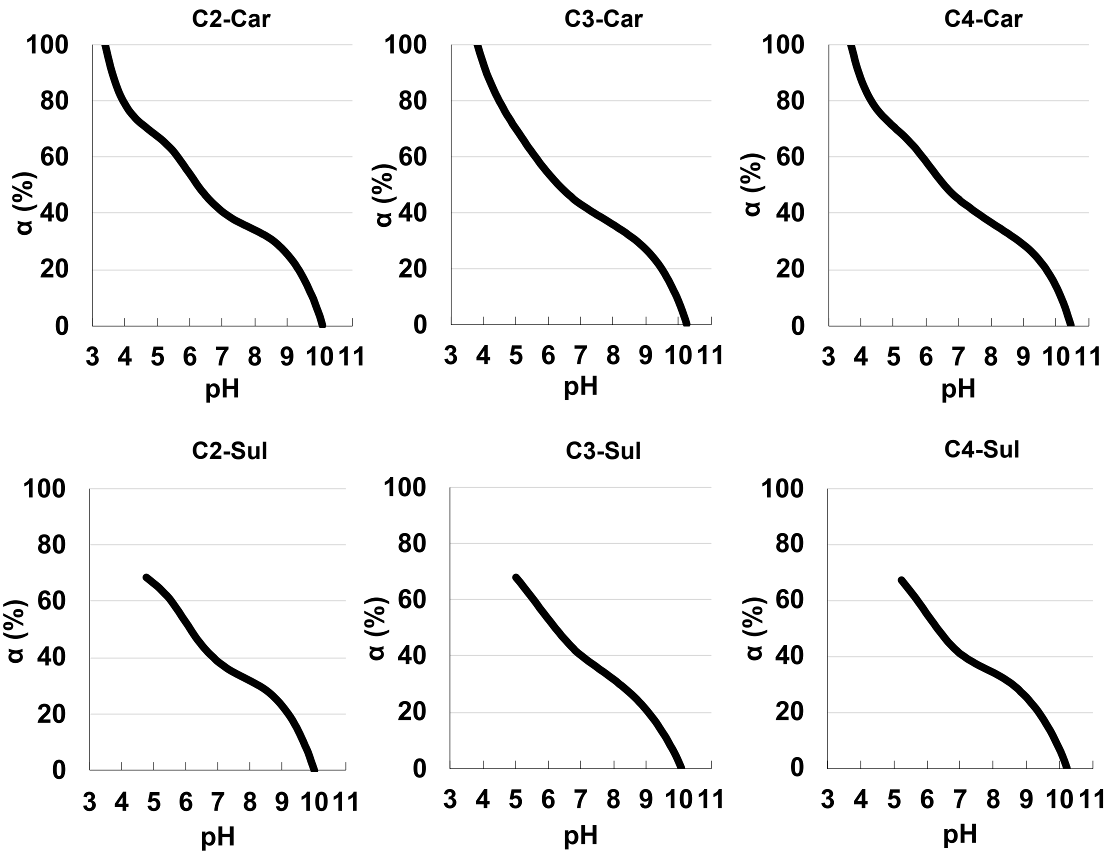

Figure S8. α/pH curves of C*n*-Car and C*n*-Sul.

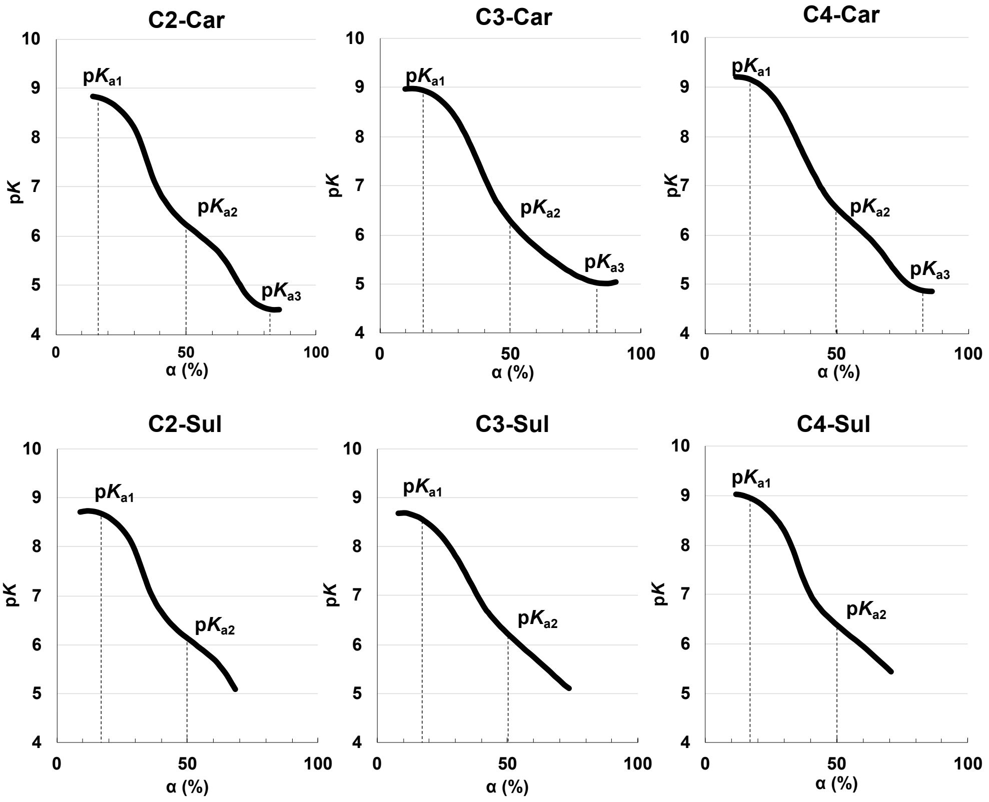

Figure S9. pK/α curves of Cn-Car and Cn-Sul.

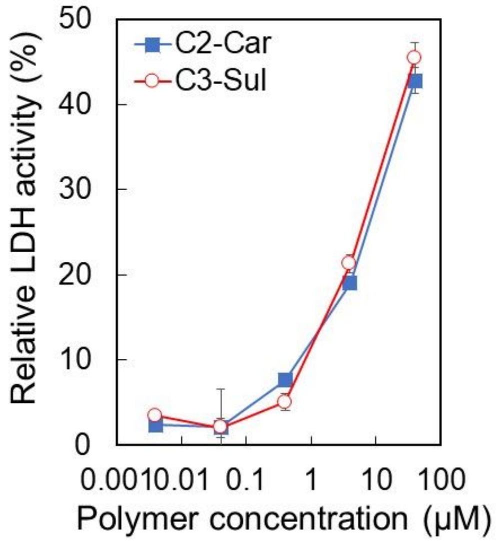

Figure S10. Activity of lactase dehydrogenase (LDH) released from cultured Huh-7 cells after 4 h treatment with C2-Car, C3-Sul at pH 5.5. The results represent the mean ± SEM (n = 6).

Computational Parameters

Table S1. Calculated electronic energy, enthalpy, and Gibbs free energy (Hartree)

compound	Electronic Energy	Enthalpy	Free Energy
H_2O	-76.440343	-76.414985	-76.436401
H_3O^+	-76.833209	-76.793961	-76.793961
CAR_C2_a	-536.358010	-536.101487	-536.155614
CAR_C2_b	-536.348570	-536.091720	-536.144632
CAR_C2_c	-536.811899	-536.539409	-536.592417
CAR_C3_a	-575.672912	-575.386886	-575.443973
CAR_C3_b	-575.669490	-575.382270	-575.436083
CAR_C3_c	-576.134370	-575.832429	-575.887721
CAR_C4_a	-614.984514	-614.668530	-614.728547
CAR_C4_b	-614.983864	-614.666797	-614.723990
CAR_C4_c	-615.448766	-615.116526	-615.173258
SUL_C2_a	-971.624802	-971.365201	-971.422153
SUL_C2_b	-971.624340	-971.364695	-971.420206
SUL_C2_c	-972.075552	-971.799919	-971.854530
SUL_C3_a	-1010.942714	-1010.653270	-1010.713415
SUL_C3_b	-1010.943182	-1010.653167	-1010.710092
SUL_C3_c	-1011.402262	-1011.096741	-1011.153514
SUL_C4_a	-1050.253834	-1049.933714	-1049.994456
SUL_C4_b	-1050.255203	-1049.934922	-1049.994197
SUL_C4_c	-1050.710618	-1050.3746553	-1050.433582

Coordination

H_2O

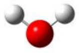

Center Number	Atomic Number	Atomic Type	Coordinates (Angstroms) X	Y	Z
1	8	0	-0.000000	0.000000	0.117302
2	1	0	0.000000	0.759306	-0.469207
3	1	0	-0.000000	-0.759306	-0.469207

H_3O^+

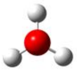

Center Number	Atomic Number	Atomic Type	Coordinates (Angstroms) X	Y	Z
1	8	0	0.000000	-0.000079	0.082426
2	1	0	0.000000	0.925255	-0.219840
3	1	0	0.801780	-0.462588	-0.219815
4	1	0	-0.801780	-0.462588	-0.219815

CAR_C2_a

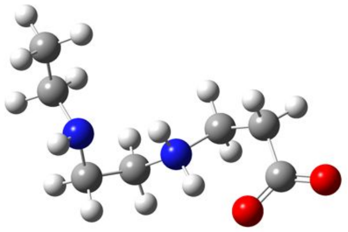

Center Number	Atomic Number	Atomic Type	Coordinates (Angstroms) X	Y	Z
1	7	0	2.334184	-0.283822	-0.698266
2	6	0	3.351451	0.330320	0.155133
3	6	0	3.840514	1.639361	-0.441743
4	6	0	1.818744	-1.555885	-0.207343
5	6	0	0.730580	-1.302251	0.833097
6	7	0	-0.238683	-0.338686	0.262873
7	6	0	-1.097668	0.374620	1.244882
8	6	0	-2.221639	1.098775	0.516549
9	6	0	-3.095371	0.192557	-0.377702
10	8	0	-4.258079	0.563730	-0.601919
11	8	0	-2.543666	-0.851663	-0.843686
12	1	0	2.701919	-0.400902	-1.634058
13	1	0	4.199774	-0.352619	0.316073
14	1	0	2.909070	0.521447	1.137698
15	1	0	4.595567	2.094431	0.203050
16	1	0	3.013877	2.344711	-0.558145
17	1	0	4.292936	1.475914	-1.424523
18	1	0	1.389602	-2.103142	-1.049778
19	1	0	2.592075	-2.192173	0.243224
20	1	0	0.204604	-2.217777	1.101492
21	1	0	1.143715	-0.860194	1.741460
22	1	0	0.323955	0.319515	-0.294608
23	1	0	-0.948833	-0.791921	-0.376123
24	1	0	-1.496988	-0.382460	1.922044

25	1	0	-0.473421	1.060197	1.818439
26	1	0	-2.856936	1.594708	1.250907
27	1	0	-1.805120	1.886308	-0.121798

CAR_C2_b

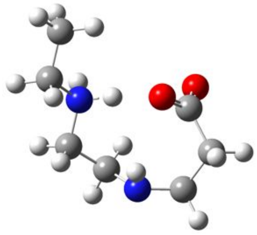

Center Number	Atomic Number	Atomic Type	Coordinates (Angstroms) X	Y	Z
1	7	0	-1.575400	0.368195	0.321043
2	6	0	-2.889669	0.249543	-0.369458
3	6	0	-3.478955	-1.133646	-0.170014
4	6	0	-0.971843	1.733850	0.259510
5	6	0	0.506163	1.714697	0.624136
6	7	0	1.361725	1.467522	-0.539052
7	6	0	2.595962	0.730781	-0.246495
8	6	0	2.514288	-0.777193	-0.554990
9	6	0	1.265338	-1.391443	0.074832
10	8	0	1.323524	-1.835471	1.233427
11	8	0	0.205730	-1.333631	-0.636197
12	1	0	-1.694554	0.113168	1.301583
13	1	0	-0.897197	-0.358439	-0.076999
14	1	0	-2.706420	0.456654	-1.424285
15	1	0	-3.543477	1.028114	0.026503
16	1	0	-4.428927	-1.200366	-0.701801
17	1	0	-3.667801	-1.334761	0.887276
18	1	0	-2.810811	-1.903915	-0.560343
19	1	0	-1.115388	2.111045	-0.754780
20	1	0	-1.538765	2.368489	0.939663
21	1	0	0.759410	2.685430	1.059943
22	1	0	0.661189	0.969588	1.418597
23	1	0	0.842518	0.953746	-1.241325
24	1	0	3.422544	1.155017	-0.824549
25	1	0	2.845173	0.879406	0.808457
26	1	0	2.471442	-0.914670	-1.638720

27	1	0	3.415043	-1.265241	-0.176115

CAR_C2_c

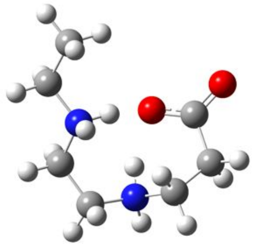

Center Number	Atomic Number	Atomic Type	Coordinates (Angstroms) X	Y	Z
1	7	0	-1.606276	-0.243101	0.241822
2	6	0	-2.843533	0.489837	-0.178124
3	6	0	-2.928370	1.829399	0.524725
4	6	0	-1.456636	-1.570771	-0.419546
5	6	0	-0.221658	-2.346266	0.007774
6	7	0	1.052163	-1.631355	-0.332368
7	6	0	1.772778	-1.012408	0.835249
8	6	0	2.571956	0.199717	0.384205
9	6	0	1.708976	1.314693	-0.234924
10	8	0	2.137336	2.466288	-0.227490
11	8	0	0.596827	0.934585	-0.753735
12	1	0	-0.773407	0.350767	-0.008013
13	1	0	-1.619826	-0.352350	1.256390
14	1	0	-3.694116	-0.149900	0.056752
15	1	0	-2.779994	0.606625	-1.259932
16	1	0	-3.826388	2.348904	0.188933
17	1	0	-2.063526	2.453062	0.288721
18	1	0	-2.993222	1.706212	1.608063
19	1	0	-2.336524	-2.167673	-0.181823
20	1	0	-1.446315	-1.389212	-1.494773
21	1	0	-0.217566	-2.563015	1.075791
22	1	0	-0.234718	-3.295461	-0.524103
23	1	0	1.683113	-2.252687	-0.835425
24	1	0	0.838323	-0.813369	-0.942766
25	1	0	1.012707	-0.721603	1.559641

26	1	0	2.397072	-1.780153	1.288467
27	1	0	3.110238	0.599779	1.243679
28	1	0	3.328560	-0.090918	-0.352141

CAR_C3_a

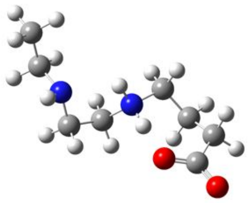

Center Number	Atomic Number	Atomic Type	Coordinates (Angstroms) X	Y	Z
1	7	0	2.594397	-0.787848	-0.168401
2	6	0	3.739445	0.062858	0.153288
3	6	0	4.611299	0.282522	-1.071871
4	6	0	1.721937	-1.081942	0.961595
5	6	0	0.743161	0.068490	1.180185
6	7	0	0.077247	0.355914	-0.111736
7	6	0	-0.650246	1.649085	-0.193587
8	6	0	-2.014581	1.582714	0.486164
9	6	0	-3.109691	0.822212	-0.273806
10	6	0	-3.052279	-0.714350	-0.302876
11	8	0	-1.909956	-1.277290	-0.284039
12	8	0	-4.132150	-1.321873	-0.380154
13	1	0	2.921137	-1.652114	-0.581886
14	1	0	4.338535	-0.368514	0.970458
15	1	0	3.365200	1.027004	0.511421
16	1	0	5.462434	0.921343	-0.826050
17	1	0	4.040808	0.758818	-1.873194
18	1	0	5.002665	-0.667686	-1.447939
19	1	0	1.158270	-1.990338	0.735555
20	1	0	2.270706	-1.256274	1.897120
21	1	0	-0.011523	-0.195751	1.920266
22	1	0	1.255455	0.973666	1.512021
23	1	0	0.814669	0.285451	-0.821228
24	1	0	-0.670660	-0.407713	-0.281635
25	1	0	-0.023194	2.418387	0.259598
26	1	0	-0.765700	1.885150	-1.253154

27	1	0	-1.908989	1.188259	1.501934
28	1	0	-2.349134	2.616972	0.601366
29	1	0	-4.082664	1.098948	0.135959
30	1	0	-3.116956	1.151687	-1.320660

CAR_C3_b

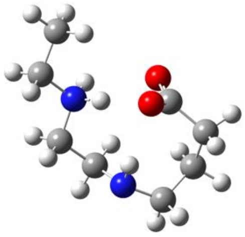

Center Number	Atomic Number	Atomic Type	Coordinates (Angstroms) X	Y	Z
1	7	0	-1.659526	0.443511	0.174481
2	6	0	-3.110240	0.432305	-0.152235
3	6	0	-3.663871	-0.979028	-0.093920
4	6	0	-1.048128	1.804767	0.191839
5	6	0	0.439288	1.766401	0.507328
6	7	0	1.238542	1.428915	-0.664993
7	6	0	2.618101	1.041594	-0.374443
8	6	0	2.831013	-0.242361	0.446609
9	6	0	2.193078	-1.502374	-0.162159
10	6	0	0.670760	-1.497082	-0.018689
11	8	0	0.187455	-1.611505	1.126804
12	8	0	-0.023955	-1.297639	-1.067887
13	1	0	-1.501655	-0.006070	1.081041
14	1	0	-1.140163	-0.211130	-0.477646
15	1	0	-3.212592	0.860122	-1.150152
16	1	0	-3.612620	1.090842	0.557663
17	1	0	-4.723546	-0.960796	-0.352033
18	1	0	-3.565790	-1.401151	0.909031
19	1	0	-3.148673	-1.633109	-0.800301
20	1	0	-1.227649	2.255795	-0.785553
21	1	0	-1.583109	2.381664	0.946512
22	1	0	0.729534	2.763019	0.853935
23	1	0	0.595039	1.081124	1.354244
24	1	0	0.799540	0.678870	-1.188005
25	1	0	3.126653	0.927647	-1.337583
26	1	0	3.106334	1.876250	0.141236

27	1	0	3.911728	-0.397074	0.529060
28	1	0	2.462112	-0.115214	1.469195
29	1	0	2.465177	-1.589515	-1.217649
30	1	0	2.578565	-2.380437	0.363285

CAR_C3_c

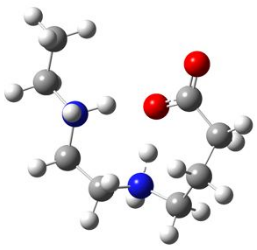

Center Number	Atomic Number	Atomic Type	Coordinates (Angstroms) X	Y	Z
1	7	0	-1.823539	-0.457382	0.330215
2	6	0	-3.176139	-0.136056	-0.220196
3	6	0	-3.603301	1.254731	0.204955
4	6	0	-1.361516	-1.842880	0.020240
5	6	0	0.095136	-2.080703	0.389407
6	7	0	1.038637	-1.516963	-0.616627
7	6	0	2.441606	-1.354598	-0.120808
8	6	0	2.617020	-0.098376	0.726950
9	6	0	2.464253	1.248215	-0.004674
10	6	0	1.028387	1.746552	-0.190290
11	8	0	0.202348	0.914074	-0.733434
12	8	0	0.725487	2.885927	0.156037
13	1	0	-1.128337	0.241637	-0.047053
14	1	0	-1.838956	-0.328561	1.342703
15	1	0	-3.865560	-0.901361	0.137190
16	1	0	-3.095682	-0.216344	-1.304433
17	1	0	-4.577907	1.476290	-0.231124
18	1	0	-2.893562	2.008296	-0.143093
19	1	0	-3.693752	1.327590	1.291041
20	1	0	-1.990217	-2.532556	0.581286
21	1	0	-1.533861	-2.015382	-1.042557
22	1	0	0.330924	-1.638787	1.357636
23	1	0	0.276292	-3.152127	0.459338
24	1	0	1.030819	-2.097005	-1.453117
25	1	0	0.707035	-0.525437	-0.870886

26	1	0	2.702310	-2.249988	0.444498
27	1	0	3.079716	-1.307952	-1.003628
28	1	0	1.962254	-0.129077	1.603466
29	1	0	3.634777	-0.154738	1.119855
30	1	0	3.008938	2.014128	0.547201
31	1	0	2.923719	1.180469	-0.997764

CAR_C4_a

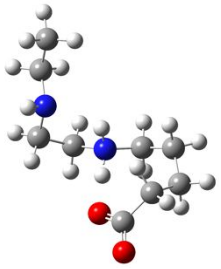

Center Number	Atomic Number	Atomic Type	Coordinates (Angstroms) X	Y	Z
1	7	0	2.502980	-0.776439	-0.395830
2	6	0	3.749597	-0.034156	-0.213258
3	6	0	4.268760	0.486062	-1.543441
4	6	0	1.951340	-1.345456	0.827744
5	6	0	1.152052	-0.286552	1.582029
6	7	0	0.178401	0.312508	0.641525
7	6	0	-0.435623	1.581538	1.095509
8	6	0	-1.337038	2.208042	0.039984
9	6	0	-2.651141	1.485125	-0.283314
10	6	0	-2.558303	0.216492	-1.166171
11	6	0	-2.688832	-1.098606	-0.389207
12	8	0	-3.686146	-1.810468	-0.580686
13	8	0	-1.750696	-1.379312	0.429265
14	1	0	2.644306	-1.509792	-1.079145
15	1	0	4.516254	-0.657243	0.273417
16	1	0	3.556632	0.809327	0.457004
17	1	0	5.198429	1.041321	-1.400644
18	1	0	3.537609	1.149508	-2.012184
19	1	0	4.473369	-0.339341	-2.232175
20	1	0	1.283438	-2.166228	0.555655
21	1	0	2.719886	-1.750518	1.500159
22	1	0	0.607485	-0.722252	2.419959
23	1	0	1.797814	0.507370	1.962918
24	1	0	0.689730	0.438664	-0.240156
25	1	0	-0.620679	-0.414704	0.477699

26	1	0	-0.997397	1.356944	2.005449
27	1	0	0.372193	2.268825	1.354457
28	1	0	-1.581837	3.206407	0.413803
29	1	0	-0.762934	2.363837	-0.881230
30	1	0	-3.175206	1.254906	0.651039
31	1	0	-3.281424	2.207161	-0.807860
32	1	0	-3.350416	0.236942	-1.914798
33	1	0	-1.607065	0.209685	-1.711105

CAR_C4_b

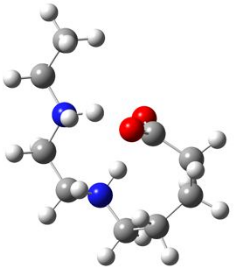

Center Number	Atomic Number	Atomic Type	Coordinates (Angstroms) X	Y	Z
1	7	0	1.902160	0.453797	0.146793
2	6	0	3.330118	0.341183	-0.245153
3	6	0	3.776127	-1.109331	-0.239620
4	6	0	1.389231	1.849716	0.215037
5	6	0	-0.092615	1.903080	0.554712
6	7	0	-0.936849	1.666182	-0.610498
7	6	0	-2.365142	1.620522	-0.293768
8	6	0	-2.859556	0.350307	0.414324
9	6	0	-2.796125	-0.919564	-0.465030
10	6	0	-1.867860	-2.017703	0.069942
11	6	0	-0.411294	-1.571041	0.136508
12	8	0	0.110778	-1.175241	-0.956557
13	8	0	0.182619	-1.574691	1.235906
14	1	0	1.295769	-0.152792	-0.483520
15	1	0	1.745946	-0.011522	1.047681
16	1	0	3.914624	0.942718	0.452848
17	1	0	3.423486	0.781690	-1.238558
18	1	0	4.822029	-1.166716	-0.544207
19	1	0	3.181690	-1.705509	-0.934888
20	1	0	3.687494	-1.545932	0.757909
21	1	0	1.973052	2.368265	0.976361
22	1	0	1.578068	2.318033	-0.752335
23	1	0	-0.286451	1.210177	1.388809
24	1	0	-0.304445	2.909371	0.928005
25	1	0	-0.674204	0.787883	-1.051361

26	1	0	-2.598374	2.497654	0.319469
27	1	0	-2.920981	1.737850	-1.230584
28	1	0	-2.286211	0.197895	1.335762
29	1	0	-3.891234	0.528357	0.732971
30	1	0	-3.798445	-1.343512	-0.570688
31	1	0	-2.472342	-0.655766	-1.477584
32	1	0	-2.187724	-2.333436	1.065855
33	1	0	-1.923398	-2.888113	-0.592513

CAR_C4_c

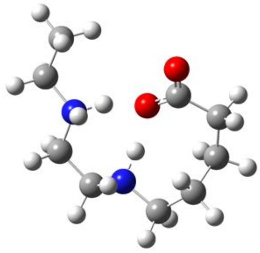

Center Number	Atomic Number	Atomic Type	Coordinates (Angstroms) X	Y	Z
1	7	0	-1.980153	-0.532902	0.288140
2	6	0	-3.315595	-0.246815	-0.314765
3	6	0	-3.756374	1.164817	0.018855
4	6	0	-1.511514	-1.929150	0.067070
5	6	0	-0.066906	-2.152539	0.488709
6	7	0	0.922197	-1.680823	-0.523428
7	6	0	2.341282	-1.713641	-0.028027
8	6	0	2.717256	-0.389358	0.621846
9	6	0	2.851524	0.777771	-0.382995
10	6	0	2.024025	2.007923	-0.022367
11	6	0	0.508481	1.817595	-0.041081
12	8	0	0.053985	0.804145	-0.696587
13	8	0	-0.210366	2.630844	0.542000
14	1	0	-1.259960	0.148609	-0.115859
15	1	0	-2.020249	-0.347172	1.290973
16	1	0	-4.016955	-0.992251	0.062206
17	1	0	-3.204580	-0.385319	-1.390693
18	1	0	-4.711511	1.364823	-0.468154
19	1	0	-3.026530	1.896342	-0.333570
20	1	0	-3.890926	1.293999	1.095200
21	1	0	-2.153418	-2.592510	0.645202
22	1	0	-1.654829	-2.160693	-0.989028
23	1	0	0.156154	-1.647743	1.429178
24	1	0	0.097568	-3.219306	0.633094
25	1	0	0.835027	-2.246059	-1.365888

26	1	0	0.689492	-0.676753	-0.774332
27	1	0	2.426535	-2.551465	0.663977
28	1	0	2.975666	-1.915769	-0.890664
29	1	0	1.987678	-0.145169	1.402140
30	1	0	3.661397	-0.550174	1.145606
31	1	0	3.901169	1.072476	-0.450851
32	1	0	2.573391	0.447312	-1.388793
33	1	0	2.296920	2.384955	0.967172
34	1	0	2.241619	2.818406	-0.726668

SUL_C2_a

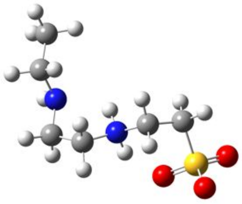

Center Number	Atomic Number	Atomic Type	Coordinates (Angstroms) X	Y	Z
1	7	0	-2.959699	-0.159812	-0.723914
2	6	0	-3.992516	0.288085	0.211824
3	6	0	-4.442064	1.701694	-0.117018
4	6	0	-2.463158	-1.509180	-0.483290
5	6	0	-1.363199	-1.459388	0.575645
6	7	0	-0.413604	-0.384056	0.185514
7	6	0	0.427766	0.152733	1.290663
8	6	0	1.521802	1.061099	0.755795
9	16	0	2.746551	0.154098	-0.219093
10	8	0	1.922545	-0.576947	-1.227015
11	8	0	3.627202	1.165858	-0.820604
12	8	0	3.425895	-0.758106	0.717650
13	1	0	-3.302763	-0.081746	-1.672917
14	1	0	-3.576652	0.263477	1.223724
15	1	0	-4.854798	-0.395356	0.205335
16	1	0	-5.210246	2.030774	0.586100
17	1	0	-4.865463	1.752253	-1.124682
18	1	0	-3.601567	2.398091	-0.062972
19	1	0	-3.241240	-2.205914	-0.147117
20	1	0	-2.049352	-1.899806	-1.415568
21	1	0	-1.765160	-1.198309	1.555658
22	1	0	-0.823082	-2.401259	0.656172
23	1	0	0.235359	-0.699177	-0.561329
24	1	0	-1.016360	0.349550	-0.225949
25	1	0	-0.224537	0.699778	1.970283
26	1	0	0.854753	-0.698161	1.823068

27	1	0	1.119395	1.847691	0.115036
28	1	0	2.053092	1.522919	1.587485

SUL _C2_b

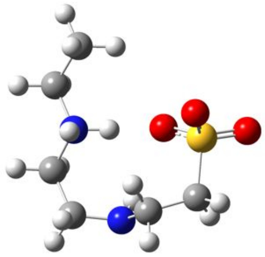

Center Number	Atomic Number	Atomic Type	Coordinates (Angstroms) X	Y	Z
1	7	0	1.923015	-0.162058	0.170597
2	6	0	2.751171	-1.342289	-0.222571
3	6	0	2.200999	-2.611832	0.397294
4	6	0	2.395763	1.128497	-0.408335
5	6	0	1.546018	2.323899	0.017863
6	7	0	0.130050	2.217492	-0.309089
7	6	0	-0.741098	1.799631	0.790129
8	6	0	-2.016107	1.149581	0.271143
9	16	0	-1.791320	-0.601266	-0.109418
10	8	0	-0.525852	-0.667237	-0.913093
11	8	0	-1.643203	-1.295918	1.182839
12	8	0	-2.965539	-1.018664	-0.889863
13	1	0	0.940450	-0.347157	-0.156384
14	1	0	1.901099	-0.097296	1.188529
15	1	0	3.775276	-1.144072	0.094318
16	1	0	2.726470	-1.385990	-1.311779
17	1	0	2.801406	-3.460239	0.066688
18	1	0	1.166976	-2.778526	0.088995
19	1	0	2.243277	-2.571506	1.488214
20	1	0	3.434303	1.271236	-0.107411
21	1	0	2.365719	1.005450	-1.492611
22	1	0	1.651799	2.499423	1.092539
23	1	0	1.986966	3.194184	-0.477988
24	1	0	-0.003977	1.611356	-1.109283
25	1	0	-0.250624	1.105055	1.489225

26	1	0	-1.014404	2.678121	1.381715
27	1	0	-2.812361	1.186043	1.014728
28	1	0	-2.366424	1.635770	-0.641561

SUL _C2_c

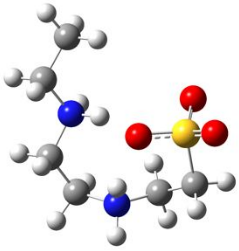

Center Number	Atomic Number	Atomic Type	Coordinates (Angstroms) X	Y	Z
1	7	0	-1.998585	0.027818	0.211734
2	6	0	-3.044847	-0.938622	-0.258102
3	6	0	-2.819846	-2.306248	0.354106
4	6	0	-2.153096	1.389950	-0.373210
5	6	0	-1.127412	2.400110	0.110528
6	7	0	0.273509	2.087232	-0.345794
7	6	0	1.242097	1.724502	0.750360
8	6	0	2.331993	0.802124	0.233144
9	16	0	1.679938	-0.854964	-0.088760
10	8	0	1.351704	-1.445615	1.213632
11	8	0	0.425138	-0.561736	-0.880517
12	8	0	2.681993	-1.570066	-0.875805
13	1	0	-2.026378	0.077745	1.230953
14	1	0	-1.063460	-0.354369	-0.065613
15	1	0	-2.969341	-0.967363	-1.345054
16	1	0	-4.014188	-0.522555	0.015276
17	1	0	-3.578770	-2.990482	-0.026718
18	1	0	-2.906447	-2.276546	1.442265
19	1	0	-1.838312	-2.702717	0.086198
20	1	0	-2.116636	1.279212	-1.457270
21	1	0	-3.141299	1.759942	-0.102461
22	1	0	-1.404780	3.369033	-0.298536
23	1	0	-1.119672	2.488224	1.195794
24	1	0	0.252800	1.275594	-0.988000
25	1	0	0.645749	2.876259	-0.873277

26	1	0	1.658755	2.649229	1.143552
27	1	0	0.678890	1.238042	1.545823
28	1	0	2.784421	1.165250	-0.690928
29	1	0	3.111184	0.698012	0.987136

SUL _C3_a

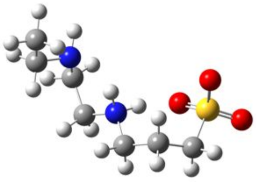

Center Number	Atomic Number	Atomic Type	Coordinates (Angstroms) X	Y	Z
1	7	0	2.914512	-0.842992	0.139285
2	6	0	4.114205	-0.018179	0.284853
3	6	0	4.503965	0.114658	1.747443
4	6	0	2.469222	-1.030250	-1.236011
5	6	0	1.613836	0.161442	-1.661534
6	7	0	0.590185	0.383401	-0.609146
7	6	0	-0.033926	1.733285	-0.609769
8	6	0	-1.143622	1.876899	0.427452
9	6	0	-2.500058	1.270535	0.065440
10	16	0	-2.640600	-0.517788	0.259040
11	8	0	-2.286805	-0.816666	1.658920
12	8	0	-4.027378	-0.854957	-0.099944
13	8	0	-1.654470	-1.105550	-0.702883
14	1	0	3.064929	-1.742523	0.578274
15	1	0	3.904441	0.973395	-0.127997
16	1	0	4.953042	-0.432930	-0.294657
17	1	0	5.398415	0.733014	1.849689
18	1	0	4.721363	-0.864862	2.183851
19	1	0	3.696883	0.574587	2.323127
20	1	0	3.296553	-1.139516	-1.949090
21	1	0	1.867859	-1.940899	-1.285520
22	1	0	2.208968	1.072459	-1.741901
23	1	0	1.112764	-0.013737	-2.612475
24	1	0	-0.174040	-0.329958	-0.686594
25	1	0	1.083789	0.198631	0.278043
26	1	0	0.761955	2.451206	-0.410272
27	1	0	-0.412409	1.912415	-1.617818

28	1	0	-0.808149	1.503022	1.399502
29	1	0	-1.302628	2.949980	0.555413
30	1	0	-3.274595	1.678990	0.716283
31	1	0	-2.774929	1.485520	-0.969468

SUL _C3_b

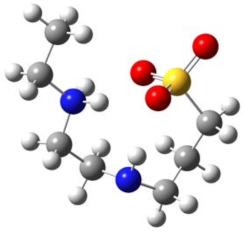

Center Number	Atomic Number	Atomic Type	Coordinates (Angstroms) X	Y	Z
1	7	0	-1.899460	0.525578	0.080545
2	6	0	-3.336981	0.147514	-0.007962
3	6	0	-3.489808	-1.360443	-0.065449
4	6	0	-1.642385	1.996395	0.111262
5	6	0	-0.184632	2.300055	0.404388
6	7	0	0.680464	1.996230	-0.727602
7	6	0	2.100498	1.937054	-0.394843
8	6	0	2.555058	0.820927	0.558708
9	6	0	2.494537	-0.609827	0.023718
10	16	0	0.875995	-1.416830	-0.007994
11	8	0	0.174325	-1.027679	1.243419
12	8	0	0.143389	-0.896010	-1.205530
13	8	0	1.151224	-2.857593	-0.110143
14	1	0	-1.459336	0.082797	0.901634
15	1	0	-1.373116	0.079814	-0.691475
16	1	0	-3.738337	0.627346	-0.901048
17	1	0	-3.833740	0.570315	0.865666
18	1	0	-4.549204	-1.611759	-0.129040
19	1	0	-3.079907	-1.833347	0.829744
20	1	0	-2.986765	-1.775279	-0.941598
21	1	0	-1.952878	2.402922	-0.852159
22	1	0	-2.277253	2.412248	0.893318
23	1	0	-0.111195	3.368251	0.630947
24	1	0	0.094458	1.763381	1.323437
25	1	0	0.421670	1.112670	-1.153747
26	1	0	2.656219	1.846968	-1.333806

27	1	0	2.385155	2.897986	0.046864
28	1	0	3.606032	1.012561	0.797787
29	1	0	2.017699	0.872400	1.509261
30	1	0	2.876111	-0.686049	-0.997620
31	1	0	3.092205	-1.266399	0.658245

SUL _C3_c

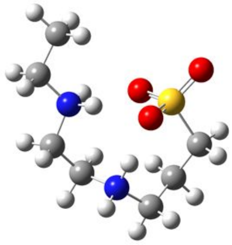

Center Number	Atomic Number	Atomic Type	Coordinates (Angstroms) X	Y	Z
1	7	0	-1.955429	0.502714	0.080689
2	6	0	-3.424147	0.234232	0.019436
3	6	0	-3.686241	-1.257707	-0.035573
4	6	0	-1.602327	1.951813	0.099611
5	6	0	-0.141383	2.198763	0.434443
6	7	0	0.788734	1.876201	-0.688821
7	6	0	2.241135	1.828705	-0.307142
8	6	0	2.608292	0.692591	0.641505
9	6	0	2.521468	-0.735951	0.104644
10	16	0	0.859604	-1.423538	-0.026460
11	8	0	1.004216	-2.858959	-0.257737
12	8	0	0.146376	-1.055351	1.220438
13	8	0	0.226637	-0.713814	-1.198516
14	1	0	-1.538582	0.029659	0.896648
15	1	0	-1.478105	0.032539	-0.705423
16	1	0	-3.803295	0.745779	-0.865160
17	1	0	-3.865612	0.690785	0.905129
18	1	0	-4.762617	-1.426830	-0.079048
19	1	0	-3.298630	-1.761337	0.852525
20	1	0	-3.235068	-1.707811	-0.922462
21	1	0	-1.887763	2.376018	-0.863358
22	1	0	-2.207902	2.421168	0.873707
23	1	0	-0.005573	3.249953	0.682025
24	1	0	0.154316	1.601161	1.294927
25	1	0	0.546086	0.942111	-1.092590

26	1	0	0.672967	2.565575	-1.431484
27	1	0	2.791398	1.741498	-1.244209
28	1	0	2.481802	2.788619	0.149108
29	1	0	3.657261	0.866472	0.893143
30	1	0	2.065643	0.766452	1.585947
31	1	0	2.964854	-0.836356	-0.888105
32	1	0	3.043421	-1.410941	0.783932

SUL _C4_a

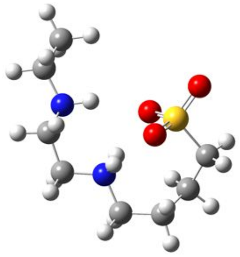

Center Number	Atomic Number	Atomic Type	Coordinates (Angstroms) X	Y	Z
1	7	0	2.584884	0.349750	-0.250454
2	6	0	3.795403	-0.408672	0.056746
3	6	0	3.986178	-1.547269	-0.932349
4	6	0	2.317072	1.394135	0.725455
5	6	0	1.213354	2.333535	0.271600
6	7	0	-0.079259	1.608571	0.139034
7	6	0	-1.249436	2.450224	-0.244193
8	6	0	-2.562734	1.681113	-0.160535
9	6	0	-2.694569	0.483697	-1.127054
10	6	0	-2.734562	-0.905750	-0.489388
11	16	0	-1.204783	-1.428963	0.305333
12	8	0	-1.062895	-0.598098	1.533091
13	8	0	-1.334188	-2.864396	0.584995
14	8	0	-0.123691	-1.105938	-0.672076
15	1	0	1.804702	-0.304358	-0.284357
16	1	0	3.771235	-0.811622	1.082911
17	1	0	4.648803	0.276229	0.006862
18	1	0	4.897282	-2.105251	-0.704365
19	1	0	4.060811	-1.165010	-1.953657
20	1	0	3.144911	-2.245664	-0.889494
21	1	0	2.072732	0.995915	1.724750
22	1	0	3.219290	2.000951	0.843129
23	1	0	1.064311	3.132302	0.998310
24	1	0	1.443932	2.768587	-0.701442
25	1	0	0.025990	0.837900	-0.539568

26	1	0	-0.305193	1.098121	1.008636
27	1	0	-1.263724	3.302427	0.435714
28	1	0	-1.062426	2.816154	-1.255066
29	1	0	-2.725613	1.360479	0.871771
30	1	0	-3.344149	2.410910	-0.381243
31	1	0	-3.629942	0.582990	-1.681710
32	1	0	-1.903792	0.505882	-1.883506
33	1	0	-3.508111	-0.978351	0.278077
34	1	0	-2.931947	-1.662048	-1.250906

SUL _C4_b

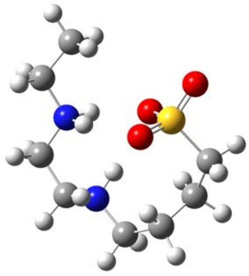

Center Number	Atomic Number	Atomic Type	Coordinates (Angstroms) X	Y	Z
1	7	0	1.913120	0.810145	0.027011
2	6	0	3.368706	0.519247	-0.089622
3	6	0	3.605141	-0.974363	-0.211157
4	6	0	1.570179	2.257013	0.128054
5	6	0	0.108037	2.458535	0.478493
6	7	0	-0.784376	2.161832	-0.635140
7	6	0	-2.196369	2.168209	-0.238324
8	6	0	-2.651996	0.897055	0.488282
9	6	0	-2.763481	-0.308829	-0.466120
10	6	0	-2.166149	-1.618555	0.035162
11	16	0	-0.361724	-1.656140	0.080011
12	8	0	0.104847	-0.972356	-1.163784
13	8	0	0.076024	-0.895009	1.284355
14	8	0	0.015450	-3.077471	0.133469
15	1	0	1.412301	0.361511	-0.757755
16	1	0	1.507029	0.305776	0.833606
17	1	0	3.849168	0.932448	0.797569
18	1	0	3.734097	1.058305	-0.964250
19	1	0	4.676596	-1.163719	-0.286379
20	1	0	3.120464	-1.379452	-1.101813
21	1	0	3.224443	-1.505607	0.664187
22	1	0	2.201867	2.676702	0.911090
23	1	0	1.824500	2.725206	-0.823578
24	1	0	-0.115803	1.871602	1.382814
25	1	0	-0.016247	3.511796	0.746131

26	1	0	-0.568333	1.248085	-1.025880
27	1	0	-2.360114	3.045345	0.395007
28	1	0	-2.807880	2.311314	-1.135702
29	1	0	-1.948644	0.676469	1.296114
30	1	0	-3.620140	1.081547	0.962531
31	1	0	-3.816381	-0.503303	-0.687017
32	1	0	-2.299321	-0.079061	-1.430222
33	1	0	-2.490472	-1.869534	1.047466
34	1	0	-2.435713	-2.442219	-0.628118

SUL _C4_c

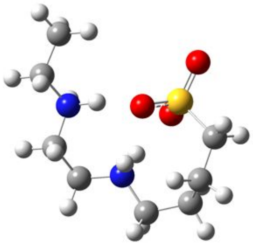

Center Number	Atomic Number	Atomic Type	Coordinates (Angstroms) X	Y	Z
1	7	0	-2.339218	0.527543	0.155674
2	6	0	-3.596343	-0.021525	-0.453740
3	6	0	-3.827165	-1.446859	0.006081
4	6	0	-2.014538	1.908858	-0.308503
5	6	0	-0.785509	2.515365	0.351453
6	7	0	0.410429	1.645974	0.186233
7	6	0	1.746813	2.312530	0.151490
8	6	0	2.853811	1.262140	0.163422
9	6	0	2.767603	0.204158	-0.962098
10	6	0	2.450882	-1.232830	-0.536504
11	16	0	0.819561	-1.535386	0.162653
12	8	0	0.756668	-0.778426	1.445646
13	8	0	-0.159583	-0.929457	-0.803697
14	8	0	0.673758	-2.981325	0.316147
15	1	0	-2.432124	0.503712	1.172400
16	1	0	-1.559862	-0.120339	-0.104053
17	1	0	-3.465510	0.035949	-1.534114
18	1	0	-4.410474	0.641334	-0.161987
19	1	0	-4.734044	-1.826465	-0.465708
20	1	0	-3.960508	-1.500833	1.088761
21	1	0	-2.995453	-2.093013	-0.282599
22	1	0	-1.883708	1.851056	-1.389700
23	1	0	-2.873810	2.544533	-0.100399
24	1	0	-0.587023	3.470433	-0.133330
25	1	0	-0.927225	2.691873	1.416739

26	1	0	0.443886	0.918312	0.929435
27	1	0	0.310696	1.091649	-0.672243
28	1	0	1.773001	2.923776	-0.751290
29	1	0	1.811731	2.963237	1.022848
30	1	0	3.791144	1.813285	0.073819
31	1	0	2.873665	0.774492	1.140952
32	1	0	2.071324	0.512251	-1.749540
33	1	0	3.738261	0.146414	-1.458028
34	1	0	2.514730	-1.900984	-1.396827
35	1	0	3.149773	-1.586486	0.224701
